# Advances in cellular and molecular mechanisms of trauma-induced organ inflammation and dysfunction

**DOI:** 10.1093/burnst/tkaf074

**Published:** 2025-11-20

**Authors:** Jieyan Wang, Hui Liang, Jie Fan

**Affiliations:** Department of Urology, People's Hospital of Longhua, No. 38 Jinglong Jianshe Road, Shenzhen 518109, Guangdong, China; Department of Urology, People's Hospital of Longhua, No. 38 Jinglong Jianshe Road, Shenzhen 518109, Guangdong, China; Department of Surgery, University of Pittsburgh School of Medicine, 3550 Terrace Street, Pittsburgh, PA 15213, United States; Research and Development, Veterans Affairs Pittsburgh Healthcare System, University Drive C, Pittsburgh, PA 15240, United States; Department of Immunology, University of Pittsburgh School of Medicine, 3550 Terrace Street, Pittsburgh, PA 15213, United States; McGowan Institute for Regenerative Medicine, University of Pittsburgh, 450 Technology Drive, Pittsburgh, PA, 15219, United States

**Keywords:** Trauma-induced organ inflammation, Molecular mechanisms, Damage-associated molecular patterns, Tramatic brain injury, Thoracic trauma, Abdominal and pelvic trauma, Extremity trauma

## Abstract

Trauma represents a significant global health issue, often resulting in devastating and long-lasting effects on the body throughout a patient's life. Organ inflammation and dysfunction caused by trauma present additional challenges for clinicians. Therefore, understanding the cellular and molecular mechanisms of post-trauma systemic inflammation and organ dysfunction is essential for improving the management of trauma. This review aims to summarize current updates on the findings that explore different mechanisms of trauma-induced inflammation and organ dysfunction, highlighting the recent understanding of the vital roles of damage-associated molecular patterns, trauma-induced cell death, organ–organ cross-talk pathways, and the gut microbiota in the development and progression of post-traumatic systemic inflammation. We also discuss new approaches that can potentially guide further investigations of trauma diagnosis, treatment, and prognosis.

## Background

Trauma refers to physical injuries caused by unexpected events that threaten life suddenly, such as bleeding and fractures [1]. Trauma is one of the leading causes of death or disability in the working-age population; however, the mortality rate in older adults is significantly higher [2], depending on complications and sequelae [3]. Fifty percent of trauma-related deaths occur within minutes, 30% of trauma patients die within 2 days because of postinjury neurological dysfunction, and 20% of patients commonly develop systemic inflammatory response syndrome (SIRS) or multiple organ dysfunction syndrome (MODS) [4]. Regarding the latter, this patient population is well suited for prophylactic interventions to prevent SIRS/MODS because of the time lag before organ inflammation and failure onset. However, in clinics, trauma is complex and requires immediate intervention provided from multidisciplinary medical expertise [5, 6]. Thus, understanding the mechanisms of trauma-induced organ inflammation and dysfunction is critical for preventing the development of traumatic death. This review focuses on current advances in trauma research, particularly the new mechanisms underlying trauma-induced organ inflammation and dysfunction. This information may provide new evidence for therapeutic strategies in trauma.

## Review

### Major types of trauma that induce organ inflammation and dysfunction

Multiple types of trauma occur according to the injury site, and can be classified as traumatic brain injury (TBI), thoracic trauma, abdominal or pelvic trauma, or extremity trauma [7]. Different types of trauma can occur alone or simultaneously as a result of different dangerous factors, leading to complex pathological mechanisms.

### TBI

TBI is defined as an external force-caused alteration in brain function or other evidence of brain pathology [8]. Traffic accidents, falls, violence, and other causes, such as sports injuries, are common causes of TBI [9]. TBI not only causes high mortality but also leads to long-term cognitive and behavioral consequences for survival [10]. Although the outcomes of TBI have improved in the past 20 years, the risk of complications and long-term mortality continues to increase [11].

### Thoracic trauma

Chest trauma is a common type of trauma, ranging from simple rib fracture to heart penetrating injury or disruption of tracheobronchial. It accounts for 25% of all traumatic deaths and is second to TBI [12]. Patients with chest trauma typically exhibit symptoms such as hypotension, shallow and decreased breathing, unstable chest walls, or subcutaneous emphysema [13]. These symptoms may indicate potential lethal injuries, including myocardial contusion, aortic disruption, pulmonary contusion, diaphragmatic rupture, and tracheobronchial and esophageal disruptions [14].

### Abdominal and pelvic trauma

Several clinical observations revealed that the mortality rate of abdominal and pelvic traumatic injuries was approximately 19% [15, 16]. Abdominal and pelvic trauma encompasses a wide array of injuries. The use of computed tomography (CT) increases diagnostic accuracy, helping patients avoid unnecessary surgical intervention and ultimately reducing morbidity [17]. The primary causes of death are uncontrolled bleeding and physiological exhaustion [18]. Intra-abdominal and pelvic adhesions are a significant complication following trauma. Many factors, including peritoneal fluid, neutrophils, macrophages, cytokines, mesothelial cells, and coagulation factors, contribute to subsequent organ dysfunction [19].

### Extremity trauma

Traumatic injuries to the extremities usually cause bleeding and fractures with complex bone, muscle, and skin injuries [20, 21]. For profuse bleeding, immediate surgical exploration is necessary. However, in less severe injuries, vascular injury occasionally still occurs, whereas open fractures are at risk of infection [22]. Evidence indicates that multi-extremity trauma induces severe inflammation and the development of soft tissue infection, posttraumatic osteoarthritis, osteomyelitis, and nonunion [23]. Recent research has revealed that neuroinflammatory reactions induced by multiple types of injury trigger the rapid recruitment and activation of immune cells and the production of chemokines/cytokines (CCl2, CCl3, CCl4, IL-1β, and IL-6), leading to secondary brain injury [24].

### Mechanisms of trauma-induced inflammation and organ dysfunction

After experiencing a traumatic event, TBI and/or hemorrhagic shock can lead to early mortality. Most patients survive resuscitation but may experience systemic inflammation and organ dysfunction [25]. Below, we summarize the current understanding of the multifactorial mechanisms that contribute to post-trauma inflammation and organ dysfunction.

### Primary mechanisms

#### Danger theory

Danger theory was first proposed by Polly Matzinger in 1994 [26]. This theory suggests that the immune system is focused primarily on detecting and protecting against endogenous danger signals (i.e. molecules released by stressed or damaged cells) rather than simply distinguishing between self and non-self. Supporting this idea, various molecules associated with tissue damage or cell death, termed danger-associated self-molecules, have been shown to trigger activation of immunity and inflammation response. These endogenous danger molecules were renamed as danger-associated molecular patterns (DAMPs) by Walter Land in 2003 [27].

DAMPs released from damaged tissue or dead cells can activate receptors, either membrane-bound or intracellular, in neighboring cells, leading to local inflammation. DAMPs can also initiate systemic inflammatory responses by reaching distant tissues or organs and mediate the interaction between different tissues or organs [28], which is crucial for tissue repair and regeneration.

**Figure 1:**
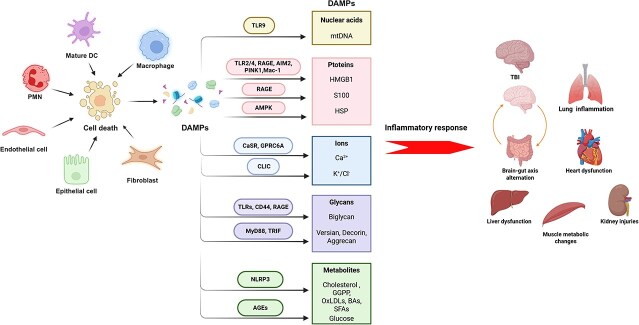
DAMP theory in trauma. DAMPs can be released by various cells, such as macrophages, DCs, neutrophils, epithelial cells, endothelial cells, and fibroblasts, to induce the expression and release of inflammatory cytokines and chemokines and immune responses. DAMP molecules are typically divided into five categories: nucleic acids, proteins, ions, glycans, and metabolites. MtDNAs are the major nucleic acids released by damaged cells into the cytoplasm. Dying cells also release DAMP proteins, such as redox-sensitive HMGB1, S100, and HSP proteins. Ca^2+^, K^+^, and Cl^−^ channels are three important cellular ion DAMPs. These channels activate the NLRP3 inflammasome, leading to immune responses. Glycans include biglycan, versican, decorin, and aggrecan. Glycans are located on the outer membrane of cells and initiate immune responses. The accumulated metabolites from lipid and glucose metabolism are converted into DAMPs under damage conditions. For example, GGPP, OxLDLs, cholesterol crystals, and BAs are derived from cholesterol metabolism, primarily SFAs from fatty acid metabolism, and glucose and citrate from glucose metabolism. *DC* dendritic cells, *TLR* toll-like receptor, *RAGE* receptor for advanced glycation-end products, *AIM2* absent in melanoma 2, *PINK1* PTEN-induced putative kinase 1, *Mac-1* macrophage-1 antigen 1, *AMPK* adenosine 5′-monophosphate (AMP)-activated protein kinase, *DAMPs* damage-associated molecular patterns, *CaSR* calcium-sensing receptor, *GPRC6A* G protein-coupled receptor family C group 6 member A, *MyD88* myeloid differentiation factor-88, *TRIF* TIR domain-containing adaptor-inducing IFN-beta, *NLRP3* NLR family pyrin domain-containing 3, *AGEs* advanced glycation end products, *mtDNA* mitochondrial DNA, *HMGB1* high-mobility group box protein 1, *Ca*  ^*2+*^calcium, *K*  ^*+*^ potassium, *Cl*  ^*-*^chloride, *GGPP* geranylgeranyl diphosphate, *OxLDLs* oxidized low-density lipoproteins, *Bas* bile acids, *SFAs* saturated fatty acids, *TBI* traumatic brain injury. This figure was created with BioRender. https://BioRender.com/ruijymn

Over the past two decades, many DAMPs, including nucleic acids, proteins, ions, glycans, and metabolites, have been identified [29]. Studies have shown that cells can sense DAMPs deriving not only from the same cell but also from neighboring cells, as well as from distant tissues or organs [30]. Intracellular DAMPs can be recognized by intracellular receptors, including toll-like receptors (TLRs), purine receptor 7 (P2X7), receptors for advanced glycation end products (RAGE), and the NOD-like receptor family (NLRs), in a variety of diseases, triggering downstream inflammatory cascades [31, 32] (Figure 1).

Released nucleic acids serve as important DAMPs. Nucleic acids are released when nuclear or mitochondrial membranes are disrupted by traumatic cell damage and subsequently activate immune responses. For example, during trauma-induced organ failure and immunoparalysis, massive amounts of mitochondrial deoxyribonucleic acid (mtDNA) are released into the cytoplasm [33]. This mtDNA is recognized by Toll-like receptor 9 (TLR9) and induces the synthesis of IFN-α and other proinflammatory cytokines via the mitogen-activated protein kinase (MAPK), nuclear factor-kappa B (NF-κB), NLR family pyrin domain containing 3 (NLRP3) signaling pathways, or cyclic guanosine monophosphate-adenosine monophosphate synthase (cGAS) [34]. DAMP proteins, such as redox-sensitive S100, heat shock protein (HSP), and high-mobility group box 1 (HMGB1), constitute another category of important DAMPs [35]. For instance, HMGB1 acts via Toll-like receptors-2/4 (TLR2/4) or interacts with RAGE and Mac-1 (macrophage 1 antigen) to induce NF-κB-dependent transcription, leading to the recruitment of immune cells and the activation of Th2 immunity [36]. HMGB1 also binds to the DNA-binding site of absent in melanoma 2 (AIM2) and activates the AIM2 inflammasome to trigger proinflammatory autophagy and apoptosis during trauma [37]. Deng *et al.* reported that HMGB1 can also act through RAGE on macrophage to trigger HMGB1 endocytosis, which subsequently initiates lysosome rupture and cathepsin B activation and release, followed by the formation of pyroptosome and activation of caspase-1, which serves as a mechanism of HMGB1-induced macrophage pyroptosis [38]. However, some studies have reported contradictory findings on the role of HMGB1, which presents intrinsic balancing mechanisms to modulate the immune state. The structure of HMGB1 contains three functional domains, including Box A, Box B, and a terminal acidic tail. Among them, Box B has proinflammatory effects. However, Box A interacts with Box B to suppress the immune response [39]. In a rat model, researchers reported that Box A of the HMGB1 protein enhances wound healing through the formation of stabilized DNA to prevent secondary DNA damage [40]. Furthermore, HMGB1 is highly expressed in bone tissue and promotes PINK1/Parkin-mediated mitochondrial autophagy, which is beneficial for fracture healing [41].

S100 proteins are biomarkers and are positively associated with the severity of trauma [42, 43]. Notably, S100B levels are good prognostic markers for mild TBI, and maintaining normal S100B protein levels indicates a positive outcome [44, 45]. S100B also interacts with RAGE to damage the endothelial glycocalyx by enhancing ADAM17 expression in endothelial cells (ECs) after TBI [46]. In addition, HSPs modulate cell death and inflammation in traumatic injury, especially in TBI [47]. HSP70 can be detected after 2 days of severe trauma and plays neuroprotective roles in brain trauma models. In TBI, the overexpression of HSP70 and HSP27 reduces blood–brain barrier (BBB) disruption and controls lesion size, leading to better neurological function [48]. These effects were also observed for HSP22 in early TBI. HSP22 improved oxidative stress and mitochondrial apoptosis to alleviate TBI-associated subarachnoid hemorrhage (SAH) and brain edema. During these processes, HSP22 through AMPK-PGC1α signaling modifies mitochondrial biogenesis, increases mtDNA and ATP, and blocks cytochrome c-triggered apoptosis [49].

Glycans are sited on the cells outer membrane and bind to immune receptors to initiate immune responses. Biglycan overloading is sensed by TLRs, CD44, and RAGE receptors to modulate the NLRP3 inflammasome to increase interleukin-1beta (IL-1β) activation during cell damage [50]. Other glycans, such as versican, decorin, and aggrecan, engage myeloid differentiation factor-88 (MyD88) or TIR domain-containing adaptor inducing IFN-beta (TRIF) to promote NF-κB or ERK/p38 signaling in macrophages to express TNF-α, IL-6, IL-12, C-X-C motif ligand 1 (CXCL1), and C-C motif chemokine ligand 2 (CCL2), which consequently chemoattract neutrophils, macrophages, and lymphocytes to injury areas [51].

Calcium (Ca^2+^), potassium (K^+^), and chloride (Cl^−^) are considered three important cellular ion DAMPs. K^+^ and Cl^−^ efflux activate the NLRP3 inflammasome [52, 53]^.^ Cell damage-induced Ca^2+^ overload is induced by calcium-sensing receptor (CaSR) and G protein-coupled receptor family C group 6 member A (GPRC6A), resulting in NLRP3 activation via the recognition of reactive oxygen species (ROS) and mtDNA, leading to immune responses [54].

Under damage conditions, the accumulated metabolites from lipid and glucose metabolism can be converted into DAMPs [55]. For instance, cholesterol crystals, bile acids (BAs), farnesyl pyrophosphate (FPP), oxidized low-density lipoproteins (OxLDLs), and geranylgeranyl diphosphate (GGPP) can be derived from cholesterol metabolism. Saturated fatty acids (SFAs) are primarily converted from fatty acid metabolism, and glucose, hyaluronan, and citrate are derived from glucose metabolism. These metabolite-derived DAMPs can bind to their corresponding receptors to trigger inflammation. Cholesterol crystals activate CXCL9 and CXCL10 to downregulate expression of MRC1 and CCL13 to trigger inflammation [56]. Glucose and its precursor advanced glycation end products (AGEs) drive the release of the cytokines IL-6, IL-1β, TNF-α, and sustained inflammation through the NF-κB signaling and PI3K/Akt pathway [57, 58] (Table 1).

**Table 1:** Different DAMPs in traumatic injuries

DAMPs	Receptors	Outcome	Ref
mtDNA	TLR9	Pro-inflammatory NF-κB, MAPK/cGAS NLRP3 activation	[33]
HMGB1	TLR2/4, or RAGE, Mac-1	NF-κB and activates Th2 immunity	[35]
	AIM2	Activate AIM2-inflammasome	[36]
	RAGE	Cathepsin B and caspase-1 activation Macrophage endocytosis	[37]
	PINK1/Parkin	Mitochondrial autophagy Fracture healing	[40]
S100B	RAGE	NF-κB inflammation Intracranial haemorrhage Shedding endothelial glycocalyx by enhancing ADAM17	[42] [43] [45]
HSP70/27	Phosphorylation	Mediate BBB disruption NF-κB activation Cell apoptosis	[47]
HSP 22	AMPK-PGC1α	Oxidative stress Mitochondrial apoptosis Increased mtDNA and ATP	[48]
Biglycan	TLRs, CD44, RAGE	NLRP3 inflammasome-induced cell damage	[49]
Versican/ decorin/ aggrecan	MyD88, TRIF	Promote NF-κB or ERK/p38 signaling in macrophages Release TNF-α, IL-6, IL-12, CCL2, and CXCL1	[50]
K^+^/Cl^−^	CLIC	NLRP3 inflammasome ROS production	[51, 52]
Ca^2+^	CaSR, GPRC6A	NLRP3 inflammasome ROS and mtDNA release	[54, 56]
Cholesterol crystals	NLRP3	Trigger caspase-1 and IL-1β	[56]
Glucose	AGEs	TNF-α, IL-6, and IL-1β release NF-κB activation-induced inflammation	[57, 58]

*NF-κB* nuclear factor-kappaB, *MAPK* mitogen-activated protein kinases, *cGAS* cyclic guanosine monophosphate-adenosine monophosphate synthase, *NLRP3* NOD-like receptor family pyrin domain-containing 3, *HMGB1* high mobility group box 1, *AIM2* absent in melanoma 2, *BBB* blood brain barrier, *MyD88* Myeloid differentiation factor-88, *TRIF* TIR domain-containing adaptor interferon-beta, *TNF-α* tumor necrosis factor-Alpha, *IL-6* interleukin-6, *CCL2* C-C motif chemokine ligand 2, *CXCL1* C-X-C motif ligand 1, *CaSR* calcium-sensing receptor, *GPRC6A* G protein-coupled receptor family C group 6 member A, *Ca*^*2+*^calcium, *K*^*+*^ potassium, *Cl*^*−*^ chloride, *CLIC* chloride intracellular channels, *ROS* reactive oxygen species, *PINK1* PTEN-induced putative kinase 1

### Trauma-induced cell death

Programmed cell death, including apoptosis, necroptosis, pyroptosis, and ferroptosis, is an important pathobiological change in trauma. DAMPs and inflammatory cytokines released during trauma can induce cell death in both innate immune cells and organ parenchyma. In turn, the induced cell death exacerbates inflammation and further cell death, thereby enhancing post-trauma inflammation and organ dysfunction.

Apoptosis is a common and caspase-dependent cell death. In trauma injuries, apoptosis can be induced by the inflammatory factor IL-1β [59] and is present in different types of cells, such as neurons [60], hepatocytes [61], microglia, and cardiomyocytes [62]. Apoptosis can be activated by numerous factors, including injury-induced calcium overload, free radicals, mTORC1, nitric oxide (NO), and fibroblast growth factor receptor 1 (FGFR1) [63]. Proapoptotic factors can trigger cytochrome-c-induced intrinsic mitochondrial cell death or the extrinsic death receptor CD95 (Fas)/Fas ligand (Fas L) pathway [64]. The canonical intrinsic apoptosis pathway is modulated by Bax/Bak, followed by downstream cytochrome-c (Cyto C) release and caspase 9 activation. The TNF-mediated death receptor pathway leads to the dysregulation of complex IIa receptor-interacting protein kinase (RIPK)1/3 and downstream caspase 8 activation. All of these pathways stimulate the expression of downstream caspases 3 and 7 to initiate apoptosis [65]. Studies have reported that within 1 h after trauma, caspases 8 and 9 are detected, and after 6 h, caspase 3 is present [66].

Necroptosis is orchestrated by RIPK1, RIPK3, and mixed lineage kinase domain-like protein (MLKL) proteins. Studies reported that traumatic injury induces retinal ganglion cell necroptosis, which exacerbates gliosis and neuroinflammation [67]. Studies have shown that necroptosis modulates lysosomal defects, mitochondrial dynamics, axonal degeneration, as well as inflammatory responses in TBI [68]. Some molecular signaling pathways, including the c-Jun N-terminal kinase (JNK), PI3K/Akt/GSK-3β, Wnt/β-catenin, and ERK/MAP pathways are critically involved in the process of necroptosis-induced neuronal damage [69]. Li *et al.* reported the significant role of trauma in inducing mtDNA fragmentation in macrophages and the consequent regulation of macrophage death [70]. The study used an animal pseudofracture (PF) trauma model to show that tissue damage induced nicotinamide adenine dinucleotide phosphate (NADPH) oxidase activation and increased the release of ROS through cold-inducible RNA-binding protein (CIRP)-TLR4-MyD88 signaling [70]. This subsequently activated endonuclease G, which executed the mtDNA fragmentation in macrophages. This study further revealed that mtDNA damage triggered macrophages necroptosis. However, the regulation of necroptosis following trauma and hemorrhagic shock (HS) is complicated. One study investigated the impact of trauma on lipopolysaccharide (LPS)-induced macrophage necroptosis and the mechanism using a mouse PF model [71]. These results demonstrate that the activity of LPS via TLR4 induces macrophage necroptosis. Interestingly, tissue damage through the mediation by HMGB1/RAGE signaling increases caveolin-1 expression in macrophages, thereby promoting caveolae-mediated TLR4 internalization and desensitization, ameliorating LPS-induced macrophage necroptosis. The study further demonstrated that the upregulation of caveolin-1 expression is regulated by RAGE–MyD88 signaling activation of Cdc42 and the subsequent nuclear translocation of specificity protein 1 (Sp1). This study reveals the protective role of DAMPs in limiting inflammation in response to pathogen-associated molecular pattern (PAMP) molecules.

Pyroptosis is a programmed and proinflammatory form of cell death regulated by the gasdermin protein family (mainly gasdermin D and GSDMD) through caspase-1 [72]. Usually, this cell death process results in the formation of pores in the plasma membrane and the release of cellular contents, including DAMPs and inflammatory cytokines such as IL-18, which trigger inflammation [73]. *In vitro* and *in vivo* experiments revealed increased caspase-1 and GSDMD expression levels in trauma injuries [74]. Ge *et al.* reported that NLRs and AIM2 inflammasomes mediate pyroptosis to worsen BBB damage following TBI [75]. CIRP, an important DAMP, also induces pyroptosis. One study reported that administration of recombinant murine CIRP (rmCIRP) in C57BL/6 mice through intravenous injection causes lung injury, which is accompanied by the assembly of the NLRP3 inflammasome and caspase-1 activation in lung ECs and subsequent IL-1β release and pyroptosis [76]. Another study demonstrated the important role of HS in the priming of LPS-induced lung EC pyroptosis [77]. Through TLR4, LPS activates the NLRP3 inflammasome in mouse lung vascular ECs (MLVECs), which is followed by activation of caspase-1. However, the HS-induced release of HMGB1, which acts through RAGE, triggers EC endocytosis of HMGB1 and then elicits a series of intracellular changes, including lysosome rupture and cathepsin B release, which promotes the assembly of pyroptosome and activation of caspase-1. These results indicate that HS sensitizes EC responses to LPS and promotes EC pyroptosis.

HS promotes the activation of NLRP3 inflammasome in the lungs through suppressing pyrin production [78]. Pyrin is a protein with 781-amino acid and a pyrin domain (PYD) at the N-terminus. Pyrin suppresses the activation of inflammasome through interactions with NLRP. In macrophages, LPS and IL-10 can be potent inducers of pyrin expression. While LPS activates the NLRP3 inflammasome in the lungs, LPS also stimulates pyrin expression, which therefore suppresses the activation of inflammasome. Importantly, the LPS-increased IL-10 expression enhanced pyrin expression, further suppresses inflammasome activation. Nevertheless, HS through suppressing IL-10 expression in alveolar macrophages boosts the activation of inflammasome and the release of IL-1β.

By reducing pyroptosis with small peptide carbon monoxide-releasing molecule-3 (CORM-3), trauma-induced acute lung injury (ALI) and acute respiratory distress syndrome (ARDS) can be alleviated by activating PKG-ERK1/2 signaling [79]. Another peptide, ghrelin, inhibits inflammasome-mediated pyroptosis through blocking NF-κB signaling, leading to a protective effect on ALI [80].

Ferroptosis was first recognized in 2012 by Dr Stockwell and is characterized by the accumulation of iron-dependent lipid peroxides [81]. Ferroptosis is closely related to neuronal death in TBI through the regulation of glutathione peroxidase 4 (GPX4) [82]. Because iron deposition is a key pathological event in ferroptosis, some inhibitors that suppress iron overload have been suggested to decrease TNF-α, IL-1β, and caspase-3 levels [83, 84]. Recent studies have shown that in trauma, increased EC ferroptosis, neutrophil extracellular trap (NET) infiltration, maintenance in the intestinal microvasculature, and suppression of NET formation can diminish endothelial ferroptosis to improve trauma-induced intestinal injury through Fundc1-dependent mitophagy [85]. Programmed cell death plays important roles in trauma-induced inflammation and organ dysfunction (Table 2). However, communication among different modes of cell death has been reported as well. Autophagy is a cellular self-renewal process in which dysfunctional and unnecessary cellular components are recycled to sustain homeostasis. Autophagy inhibition can induce cell death after trauma [86]. After traumatic spinal cord injury, for example, the inhibition of autophagy leads to increased necroptosis and pyroptosis in nerve cells [87]. Studies have shown that autophagy agonists alleviate traumatic injury by reducing proinflammatory pyroptosis and necroptosis through AMPK signaling to restore neural function [88, 89]. Mitophagy is a special form of autophagy in mitochondria. Mitophagy is mediated by the PINK1/Parkin pathway, and the activation of mitophagy suppresses neuronal apoptosis, pyroptosis, and ferroptosis after TBI [90].

**Table 2:** Mechanisms of four primary modes of programmed cell death

Cell death	Triggers	Mechanism	Ref
Apoptosis	IL-1β	HMGB1/TLR4/SDF-1	[59]
	Injury-induced calcium overload	Bax/Bak Cyt C-induced mitochondrial cell death (Fas)/Fas ligand (Fas L)	[63]
	mTORC1		
	NO		
	TNF		
	FGFR1		
	Fas/Fas L	FADD/caspase 3/8 Bcl-2/Cyt C/caspase 9	[64]
	TNF	RIPK1/3/caspase 3/8	[65]
Necroptosis	RIPK1, RIPK3	Phosphorylation of MLKL	[67]
	PI3K/Akt/GSK-3β, JNK, ERK/MAPK, Wnt/β-catenin	TNFR1/TRADD activates RIPK1/RIPK3 complex and phosphorylation of MLKL, leading to mitochondrial fission	[69]
	CIRP, TLR4, MyD88	NADPH oxidase activation and ROS release, activate endonuclease G	[70]
	TLR4	HMGB1/RAGE/Sp1/caveolin-1	[71]
Pyroptosis	DAMPs, IL-18	GSDMD/caspase 1	[74]
	NLRs and AIM2 inflammasomes	Blood–brain barrier damage	[75]
	CIRP	NLRP3 inflammasome and caspase-1 activation, IL-1β release	[76]
	TLR4	NLRP3 inflammasome, HMGB1/RAGE/caspase-1 activation	[77]
	IL-10	NLRP3/pyrin/IL-1β secretion	[78]
	CORM-3	Activate PKG-ERK1/2	[79]
	IL-1β, IL-6, TNF-α, IL-18	NLRP3, Caspase1-P20, HMGB1 and Gasdermin D and downstream NF-κB	[80]
Ferroptosis	Aging, Fe^2+^	ROS/GPX4	[82]
	Erastin	Fenton/ROS increase and GPX4 decrease	[83, 84]
	NET	Fundc1 phosphorylation-induced mitophagy	[85]

*mTORC1* mechanistic target of rapamycin complex 1, *NO* Nitric oxide, *FGFR1* fibroblast growth factor receptor 1, *Fas* death receptor CD95, *Fas L* Fas ligand, *Cyto C* cytochrome-c, *RIPK* Receptor-interacting protein kinase, *MLKL* mixed lineage kinase domain-like protein, *JNK* c-Jun N-terminal kinase, *NADPH* nicotinamide adenine dinucleotide phosphate, *CIRP* cold-inducible RNA-binding protein, *Sp1* specificity protein 1, *GSDMD* gasdermin D, *NLRP* NOD-like receptor pyrin domain-containing protein, *PKG* protein kinase G, *CORM-3* carbon monoxide-releasing molecule-3, *IL-6* Interleukin-6, *HMGB1* High Mobility Group Box Protein 1, *TNF-α*: Tumor necrosis factor-alpha, *ROS* reactive oxygen species, *GPX4* glutathione peroxidase 4, *Fe*^*2+*^ ferrous, *NET* neutrophil extracellular trap

### Trauma-induced coagulopathy

Dysregulated anticoagulation systems are the leading mechanisms of disseminated intravascular coagulation in traumatic-induced coagulopathy (TIC). Several key factors contribute to these processes. In traumatic tissue, cell death and NETs release DAMPs into the circulation, evoking systemic inflammation. Tissue factor (TF)-bearing procoagulant extracellular vesicles are released, resulting in plasma membrane rupture and gasdermin D-mediated pore formation [91]. Inflammation-induced fibrinolysis also causes vascular endothelial (VE) injury, leading to coagulopathy. On the other hand, the interaction between inflammation and coagulation causes insufficiently regulated thrombin, and this disruption of homeostasis aggravates disseminated microvascular thrombosis, affecting the prognosis of trauma patients [92]. Another important component in the TIC mechanism is dysregulated platelet function, resulting in increased clot formation [74]. Crosstalk among tissue injury, ECs, the immune system, platelet, and clotting activation provokes and exacerbates multiple organ dysfunction [93]. In addition, the TF expressed on the surface of epithelial cells provides hemostatic protection to the brain, heart, and other vital organs. Under traumatic conditions, TF triggers both arterial and venous thrombosis and generates coagulation proteases involved in TIC [94].

In TIC, ECs generate thrombin and enhance the protease-activated, receptor-mediated bidirectional interplay between inflammation and coagulation [91]. Low levels of disintegrin-like and metalloprotease with thrombospondin type 1 motif 13 (ADAMTS13) are associated with coagulopathy, EC damage, and trauma mortality [95]. Conversely, high sCD40L expression in the early stage of trauma is associated with tissue and endothelial damage, enhanced shock, coagulopathy, sympathoadrenal activation, and mortality [96]. Endothelial glycocalyx damage is another emergency inflammatory response in traumatic coagulopathy. Traumatic shock causes the loss of the glycocalyx of ECs, exposing VE cadherin, leading to disruption of endothelial gap junctions. These alterations are followed by leucocytes recruitment and activation in the site of infection and damage, where they contribute to lactic acidemia and systemic inflammation [97]. In addition to leucocytes, neutrophils are also recruited and bind to activated platelets to form NETs via the HMGB1 pathway. Once NETs are released, extracellular pathogens in blood and damaged tissues are trapped and killed [98]. Some studies have reported that neutrophil–platelet aggregates damage the endothelial barrier and cause a shift in the procoagulant phenotype of ECs [96]. However, other reports have indicated that NETs also promote the hypercoagulable state in trauma patients [99]. CXCR1/2-stimulated NETosis is highly expressed in animal models and patients and accelerates thrombin generation. The inhibition of CXCR1/2 ligands reduces NET formation, multiorgan injury, and mortality [100, 101]. Thus, rapidly correcting coagulation abnormalities represent a potential approach to improve trauma injuries.

### Secondary consequences

#### Trauma-induced immunometabolism dysfunction

Recent studies have shown that mitochondrial metabolic pathways not only provide energy for cells but also reprogram the proinflammatory and anti-inflammatory machinery in immune cells as a secondary consequence following trauma. The immunometabolic axis includes metabolic pathways, including glycolysis, glutaminolysis, pentose phosphate, and lipid oxidation, which play critical roles in inflammation [102]. Hyperglycemia is common in patients after TBI and is an indicator of damage severity. During damage, various proinflammatory cytokines, such as IL-1, IL-6, and TNF-α, activate the hypothalamic–pituitary–adrenal (HPA)-axis and insulin resistance via the NF-κB signaling pathway [103], leading to high levels of glucose and disruption of the BBB and neuronal apoptosis [104].

Mitochondria are important organelles associated with energy production. Early TBI induces mitochondrial dysfunction, along with increased ROS levels and decreased ATP production. Xu *et al.* reported that mitochondrial fission is harmful to neurons, microglia, and astrocytes. In addition, by controlling mitochondrial metabolism via adenosine 5′-monophosphate (AMP)-activated protein kinase (AMPK)/silent information regulator 1 (SIRT1)/peroxisome proliferator-activated receptor gamma coactivator 1-alpha (PGC-1α) signaling, melanocortin 1 receptor (MC1R) attenuated brain damage [105].

Altered lipid metabolism is a potential marker of trauma. The loss of all classes of circulating lipids was observed in the early stage in post-trauma patients, followed by delayed and selective lipogenesis. This alteration usually predicts worse patient outcomes [106]. Lipid metabolites, such as OxLDLs and fatty acids, activate the traditional sensing receptors TLR2, TLR4, and NLRP3. These factors aggravate inflammation and traumatic thrombosis through the MAPK-NF-κB and AMPK signaling pathways [107].

Lactate metabolism is another important factor in trauma. Early in 2014, Matthew demonstrated that elevated lactate concentrations were associated with poor prognosis in trauma [108]. Recent clinical observations have also indicated that higher lactate concentrations are associated with higher mortality in patients with traumatic hemorrhage [109]. Gallagher *et al.* demonstrated that lactate is used as an energy substrate during the activation of the living brain generated by astrocytes. However, in TBI, the level of lactate predominates over its uptake. This induces the inhibition of glycolysis, leading to the aggravation of brain injury [110, 111].

### Traumatic brain injury-induced organ-organ crosstalk

**Figure 2:**
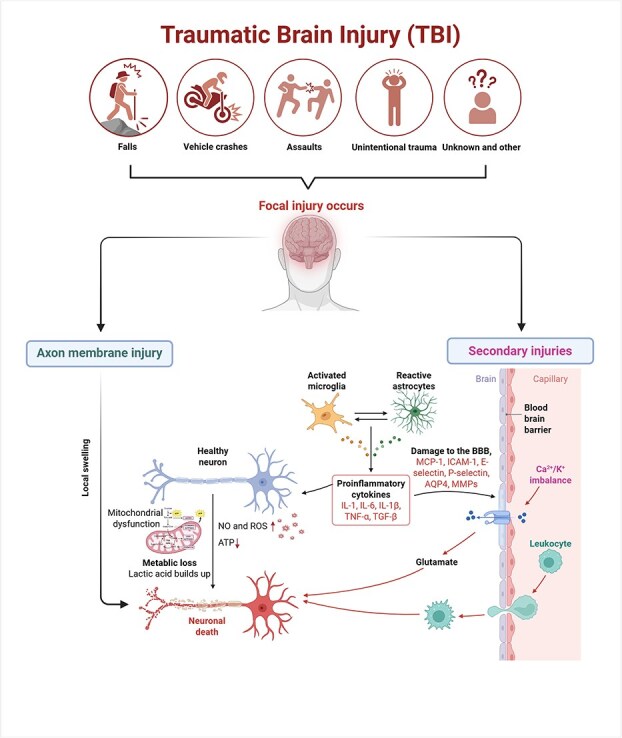
Pathology of TBI. Traumatic forces induce axon membrane injury within the white matter tracts of the brain, resulting in local brain swelling. Several hours or days later, activated microglia and reactive astrocytes release proinflammatory cytokines, such as IL-1, IL-6, IL-1β, TNF-α, and TGF-β, which breakdown multiple signaling pathways in the BBB, including MCP-1, ICAM-1, E-selectin, P-selectin, AQP4, and MMPs, resulting in secondary injury. An imbalance in Ca^2+^/K^+^ channels induces glutamate release, leading to Ca^2+^ overload in mitochondria. This triggers cellular hypoxia, lactic acid accumulation, increased ROS and NO levels, and reduced ATP synthesis. These changes decrease cerebral blood flow, followed by brain edema, an inflammatory response, and subsequent diffuse neuronal cell death. Ultimately, secondary injuries can last hours to years and are associated with dysfunctions of distant organs, such as the liver, heart, kidney, and lungs. *TBI* traumatic brain injury, *NO* nitric oxide, *ROS* reactive oxygen species, *ATP* adenosine 5′ triphosphate, *IL-6* interleukin-6, *IL-1β* interleukin-1beta, *TNF-α* tumor necrosis factor-alpha, *TGF-β* transforming growth factor-beta, *MCP-1* monocyte chemoattractant protein-1, *ICAM-1* intracellular adhesion molecule-1, *AQP4* aquaporin 4, *MMPs* matrix metalloproteinases, *Ca*^*2+*^ calcium, *K*^*+*^ potassium. This figure was created with BioRender. https://BioRender.com/8sqgjpu

Neuronal damage is prevalent after TBI injury and can disturb communication between brain regions, leading to various symptoms, such as mood, cognitive, memory, and anxiety disorders and behavioral and motor function deficits [112, 113]. Studies have revealed a cascade of cellular and molecular mechanisms that exacerbate neuronal damage, including axonal injury, BBB disruption [114], calcium efflux [115], oxidative stress, neuroinflammation [116], mitochondrial dysfunction [117], and neuronal apoptosis and ferroptosis [118, 119]. Briefly, traumatic forces induce axon membrane injury within the white matter tracts of the brain, resulting in local brain swelling; this is referred to as the primary injury. Then, local swelling progresses to secondary injury, which disrupts the balance of K^+^/Ca^2+^ homeostasis. This leads to Ca^2+^ overload in mitochondria, which triggers cellular hypoxia, lactic acid accumulation, and BBB breakdown [120]. These changes ultimately decrease cerebral blood flow [113], followed by brain edema, an inflammatory response, and subsequent diffuse neuronal cell death [121]. Secondary injuries can last hours to years and are associated with poor outcomes of TBI (Figure 2).

The BBB, formed by cerebral microvascular ECs and cell–cell tight junctions, is an important selective barrier [121]. BBB rupture is the main characteristic mediated by TBI-induced secondary injury. ECs interact with surrounding cells, such as leukocytes, neurons, microglia, pericytes, and astrocytes, to control the transport of molecules to protect the brain [122, 123]. Once the BBB breaks down, the loss of ECs allows activated leukocytes to transmigrate into the injured brain parenchyma to trigger microglial and astrocyte activation. Activated microglia release proinflammatory cytokines and increase the expression of E-selectin, P-selectin, and intracellular adhesion molecule-1 (ICAM-1) to increase BBB permeability, further increasing leukocyte adhesion and migration [124]. Astrocytes release matrix metalloproteinases (MMPs) and relocate aquaporin 4 (AQP4) to influence the junctions between ECs, and these processes are regulated by the upstream MAPK signaling pathway [125, 126]. AQP4 expression destroyed the homeostasis of ions and fluid in astrocytes, opened Ca^2+^ channels, and mediated brain edema in TBI [127]. The massive Ca^2+^ influx caused by BBB rupture also induces excitotoxicity. This process triggers the excessive release of glutamate to toxic levels, which is closely associated with neuronal death [128, 129].

In astrocyte mitochondria, damage leads to dysfunction of the mitochondrial electron transport chain (ETC) and oxidative phosphorylation (OXPHOS), along with increased production of NO and reactive oxygen species (ROS), oxidative stress and ultimately decreased adenosine 5′ triphosphate (ATP) formation [120, 130]. On the other aspect, oxidative stress contributes to BBB dysfunction and further promotes molecular damage, such as DNA damage, lipid peroxidation, protein oxidation, and inflammatory states that lead to excessive neuronal death [131].

The inflammatory response can be triggered after TBI through the release of DAMPs from primary injured tissues, such as HMGB1, further contributing to the production of proinflammatory cytokines, microglial activation, astrogliosis, macrophage polarization, BBB disruption, and neuronal death [132–134]. Constitutive DAMPs (cDAMPs) are particularly present in TBI inflammatory situations. Evidence has shown that the rapid release of cDAMPs induces the binding of IL-1α to IL-1R1 and subsequently initiates the downstream NF-κB, JNK, and MAPK pathways to promote the production of proinflammatory cytokines, including TNF-α and IL-6 [135, 136]. During TBI, macrophages and microglia secrete HMGB1, which binds to its receptor RAGE to mediate MAPK and NF-κB signaling pathway activity to regulate cytokine transcription in the nucleus [137]. Microglial polarization plays an important role in neuroinflammation following TBI-induced SAH. Recent breakthroughs have indicated that SAH can significantly induce an inflammatory response by shifting microglia from the M1 to the M2 proinflammatory phenotype. The NLRP3 inflammasome inhibitor takinib regulated the M1M2 phenotype transition and reduced lipid peroxidation, ROS overproduction, and oxidative damage. In this process, the TAK1–ROS–NLRP3 inflammasome axis represents a potential new therapeutic target for TBI and its complications [138]. IL-33 is quickly released by astrocytes and acting through MyD88 activates the NF-κB pathway [139]. Interestingly, some studies have indicated that IL-33 presents neuroprotective and anti-inflammatory effects through binding to suppression of tumorigenicity 2 receptor (ST2) [140, 141]. Thus, further studies are important to explore the effect of IL-33 on TBI. Nucleotides, such as ATP, are the fundamental cDAMP molecules that can be observed in TBI mouse models within 20 min post-injury [142]. Studies have shown that ATP plays a role in guiding microglial mobility by activating P2 receptors (specifically P2X and P2Y), which leads to neuroinflammation. However, an observational study involving patients with TBI demonstrated that those who took a P2Y inhibitor had a higher average TBI score, likely because of the antithrombotic properties of the inhibitor [143]. As noted with IL-33, more studies should be performed to assess the role of ATP in inflammation following TBI.

Notably, TBI-induced damage beyond the brain, such as to the heart, lungs, liver, kidney, and intestine, has been widely reported [144, 145].

Clinical observations have revealed that TBI patients commonly develop respiratory system complications, e.g. ARDS and ALI, which are important factors associated with poor clinical prognosis after TBI [146]. TBI models were used to investigate lung injury, and the results revealed that inflammatory cytokine production and neutrophil and macrophage infiltration in lung tissues significantly increased 24 h after TBI [147].

TBI also induces cardiac complications. Patients with TBI exhibit severe cardiac dysfunction, increased cardiac fibrosis, and inflammatory cell infiltration [148]. A multicenter study indicated an inverse relationship between brain–heart cross talk and TBI mortality [149]. The complex interactions between the brain and heart can be explained by two theories: the neurogenesis-induced myocardial phenomenon and the neuro-cardiac axis theory. In the case of neurogenic stunned myocardium, a cascade of inflammatory responses is triggered, which activates the autonomic nervous system. This activation leads to the excessive release of catecholamines, injuring heart muscle cells (myocytes) and dysfunction in the microcirculation [150]. The neuro-cardiac axis is defined as the neuronal circuits between the arteries, heart, brain, and immune organs, such as the spleen [151]. An experiment involving animals with TBI revealed that cardiac dysfunction leads to increased apoptosis of cardiomyocytes, along with inflammation and oxidative stress, starting three days after injury. This condition progresses to cardiac hypertrophy, fibrosis, and ventricular dilation by thirty days post-TBI. Additionally, performing a splenectomy (removal of the spleen) reduces cardiomyocyte apoptosis, decreases immune cell infiltration and cytokine production, lowers oxidative stress, and diminishes heart fibrosis following TBI [152].

Cirrhosis or impaired liver function can worsen the prognosis of TBI. The acute phase of TBI triggers inflammatory factors in the liver, including cytokines such as IL-1β and TNF-α, as well as chemokines such as CXCL1 and CXCL10. Three days after the onset of TBI, the increased infiltration of neutrophils further exacerbates liver damage through oxidative stress and mitochondrial dysfunction in hepatocytes [153]. The liver serves as a crucial metabolic hub, regulating the use of nutrients, proteins, and lipids for both the brain and the body. Additionally, it plays a key role in detoxification. Recent clinical evidence suggests that TBI can lead to pathological changes in the brain, which subsequently affect liver metabolism. These include de novo lipogenesis, disruptions in lipid and glucose metabolism, and lipid peroxidation [154]. By normalizing liver glucose and lipid levels, systemic and central inflammation can be affected by TBI [155]. Long-term TBI treatment leads to drug-induced liver injury, as indicated by elevated liver enzymes (AST and ALT), increased oxidative stress, inflammatory responses, and subsequent apoptosis meditated the HMGB1/TLR4/NF-κB signaling [156]. Therefore, improving brain–liver cross talk may improve post-TBI prognosis.

Understanding the brain–gut axis is essential for understanding gastrointestinal issues caused by TBI [157]. Like in other organs, inflammation is the main contributor to gastrointestinal injury, which damages the mucosal epithelial cell layer and adherent junctions, leading to mucosal barrier dysfunction and dysmotility [158]. Dysmotility changes the composition of microbes and their products and metabolites, whereas enhanced mucosal barrier permeability facilitates the passage of microbes or their metabolites across the mucosa to activate vagal and spinal afferents to mediate gut–brain communication [159]. TBI significantly alters the gut microbiome, leading to an increase in pathogenic bacteria while reducing the number of beneficial commensal bacteria [160]. Long-term inflammation and dysbiosis aggravate TBI-induced chronic effects and worsen the outcomes of TBI.

A clinical observational study revealed that approximately 15.9% of patients with TBI developed acute kidney injury (AKI) during the first 3 weeks of the ICU stay [161]. An inflammatory response with high IL-6 levels is strongly associated with renal dysfunction [162]. TBI enhances the adhesion of neutrophils and leads to apoptosis in tubular epithelial cells through a reduction in transepithelial electrical resistance [163]. TBI leads to the release of catecholamines, which negatively impact kidney blood flow and contribute to the development of AKI [164]. Additionally, increased oxidative stress is linked to severe kidney damage caused by TBI through the Kelch-like ECH-associated protein 1 (Keap1)-nuclear factor-erythroid factor 2-related factor 2 (Nrf2)/heme oxygenase 1 (HO-1) signaling pathway [165].

The potential mechanisms of multiorgan damage following TBI primarily involve intense inflammatory reactions, abnormal oxidative stress, and significant damage to ECs. These factors interact with one another, worsening the progression of organ dysfunction induced by TBI. Therefore, understanding these mechanisms could offer therapeutic opportunities for treating TBI and its related complications.

### Emerging concepts

#### Exosome-mediated post-trauma organ dysfunction

Exosomes are cell-released small vesicles and play an important role in trauma. The size of exosomal vesicles ranges from 30 to 150 nm and serves as carriers for many signaling molecules, including DNA, microRNAs (miRNAs), and proteins [166–168]. The exosome’s exceptional ability to cross bodily fluids but not being enzymatically depredated makes them unique for the material and signaling exchange between cells. Exosomes selectively pack contents, such as cytokines, transcription factors, adhesion molecules, and growth factors, to deliver messages to target cells.

In traumatic injuries, macrophage-released exosomes that contain miR-155 play a role in regulating macrophage polarization. In addition, DAMPs also increase miR-155 expression, which significantly inhibits the anti-inflammation M2 phenotype. Moreover, DAMPs increase IL-6, IL-1β, TNF-α, and IL-8 levels [169, 170]. Dendritic cells (DCs)-derived exosome presents TLR-ligands and Hsp70, that can modulate T-cell differentiation to control immune homeostasis [171]. The research in terms of the effect of exosomes on multiorgan dysfunction is limited. However, Murao *et al.* emphasized that exosomes from activated astrocytes deliver inflammatory mediators (such as IL-1β) to the central nervous system, lungs, cardiovascular system, liver, and kidneys in TBI [172, 173]. In another study, Jiao *et al.* reported that exosomes derived from HS-activated alveolar macrophages induced neutrophil NADPH oxidase activation and ROS production and subsequently promoted neutrophil necroptosis. These findings explored a mechanism of alveolar macrophage-PMN interaction following HS, which is responsible for augmented neutrophil death and subsequent amplified lung inflammation [174].

Exosomal contents appear to reflect spatial tissue pathology and the effect on remote organ function, providing an opportunity to identify new biomarkers of inflammatory posttraumatic damage.

### Gut microbiota and multiple organ dysfunction syndrome

The gut microbiota, including bacteria, viruses, and fungi, generates more than 1000 metabolites to stabilize intestinal homeostasis [175]. Once trauma occurs, massive amounts of inflammatory cytokines are released, followed by dysfunction of the gut barrier, leading to the passage of endotoxins and the pathological translocation of intestinal bacteria to distant organs and causing MODS [176]. It has been reported that the gut microbiota participates in the regulatory process of acute pathological injury after traumatic brain injury through the gut–brain axis and triggers systematic inflammation [177]. The exposure of the gut microbiota is significantly associated with immune cell phenotypes. For example, Wang *et al.* reported that segmented filamentous bacteria promote Th17 polarization and DC activation to increase the secretion of high-affinity IL-17 [178]. The XIIIAD3011 group family was positively correlated with C-C chemokine receptor type 2 (CCR2) on myeloid DCs [159]. *Fusobacterium nucleatum* stimulates regulatory CD4^+^T cells to induce inflammation in the intestinal epithelium [179]. IL-1β secretion induced by commensal microbiota was used as a marker to assess inflammation [180]. Another study revealed that T cells present protective roles in decreasing intestinal barrier damage and pathogenic bacterial invasion [181]. Changes in the abundance of the gut microbiota increase the levels of NF-κB, TNF-α, and IL-6 in response to increased intestinal permeability and enhance a proinflammatory process affecting systems outside the gut.

In addition, the gut microbiota, as well as its metabolites, influence remote cell metabolism to induce subsequent organ dysfunction. Amaral *et al.* reported that TBI induced an imbalance in the link between gut bacteria and hepatic lipids and that preemptive administration of *Lactobacillus helveticus* and *Bifidobacterium longum* probiotics influenced systemic glucose metabolism to counter memory impairment [182]. Under traumatic conditions, microbiota-generated lipids can trigger the activation of intestinal natural killer T (iNKT) cells and increase the recruitment of the iNKT into the liver and promote hepatocyte apoptosis, leading to liver damage [183].

### Gaining deeper insights into the mechanism of trauma-induced systemic inflammatory response syndrome using advanced methodology

New methods are being reported to enhance the understanding of the mechanisms underlying trauma-induced systemic inflammation. Recently, single-cell sequencing has been widely used to characterize the immune response. Since the first report of the study on single-cell RNA sequencing (scRNA-seq) in 2009, the scRNA-seq platforms became commercially available and feasible. The use of scRNA-seq has led to exciting discoveries, including new cellular subpopulations and signaling pathways from massive amounts of information at the microscopic level in trauma, especially in immune cell subsets and functions [184]. For instance, Chen *et al.* conducted a study on mononuclear cells isolated from mouse circulation and bone marrow after injured, as well as mononuclear cells collected from trauma patients’ circulation. They reported that changes in CD14^+^ monocytes are associated with the activation of inflammation and the suppression of histocompatibility complex class II (MHCII), which contributes to severe organ dysfunction in patients [185]. scRNA-seq has also been used to confirm MHCII gene expression in leukocytes from the blood, liver, and spleen [186]. Thus, in trauma studies, scRNA-seq can be used to obtain molecular information from a single cell of the epigenetic state, environment, and cell lineage. Spatial transcriptomics (ST) paves the way for tissue-level transcriptomics and epigenomics analysis. ST is currently being used in the study of trauma as well. This analysis can locate a single cell by identifying positional gene expression. Although ST technology has not yet been fully developed, it has been widely applied in clinical and biological research on trauma injuries [187]. A study analyzed 4937 tissue samples to determine the cell-type composition across five types, namely, astrocytes, ECs, microglia, neurons, and oligodendrocytes, confirming biologically meaningful results and identifying 50 differentiated genes [188], which is crucial for determining the spatial heterogeneity of trauma and guiding cell therapy in the clinic. Other transcriptomics studies have analyzed whole-blood leukocytes and classified them into two subtypes to identify dysfunctional immune responses [189, 190]. Li *et al.* applied proteomic and metabolomic approaches to classify the patterns of organ injury following severe injury in humans. In 142 patients, tissue-specific damage markers were observed. These markers were significantly associated with the degree of proinflammatory and endothelial damage. Fatty acid metabolites such as acylcarnitine and choline also indicate metabolic stress in trauma [191]. Taken together, the results of landscape studies offer valuable insights into immune status and serve as a critical resource for deeper investigation of the impact of trauma on immune responses.

### Perspective

Major advances have been made in the field of the study of trauma-induced disorders, including large-scale population-based studies, complex and diverse bioinformatics analyses, comprehensive genetics, and molecular research, which have identified factors that are linked to the mechanisms of organ inflammation and dysfunction induced by trauma. Studies have shown that quickly following injury to the brain, chest, abdomen, pelvis, or four limbs, the release of DAMPs and inflammatory cytokines from programmed dead cells can induce early pathophysiological and immune responses during trauma. DAMPs can also be spread to distant tissues through extracellular vesicles, triggering disordered cell metabolism and leading to systematic inflammation and organ dysfunction [192]. The resulting breakdown of the BBB, mucosal barrier, or endothelial barrier leads to ion and water imbalances. These imbalances trigger edema in the brain and remote organs, such as the liver, kidneys, and heart, leading to intense or continuous inflammatory reactions to aggravate multiple organ dysfunction. Therefore, therapeutic strategies must overcome the initial causes of trauma, limit further tissue damage, and address the systemic inflammatory consequences of trauma through immunomodulatory approaches.

Findings concerning the molecular and cellular mechanisms of trauma-induced organ failure have provided potential interventional and therapeutic strategies. For example, anti-DAMP therapies are used to control inflammation through the effects of HMGB1, histones 3 and 4, and ATP, as well as the consequences of different forms of cell death [193]. In response to the administration of HMGB1 antagonists, such as resveratrol, the HMGB1-mediated downstream inflammatory TLR4/RAGE pathway is inhibited, and HMGB1-induced cellular pyroptosis is blocked in macrophages [194, 195]. HMGB1 antagonists or inhibitory peptides affect the differentiation of macrophages and T cells (TH1/TH2 cells) [195]. HMGB1 inhibition restrained ferroptosis by activating Nrf2 and its downstream enzymes HO-1 and NADPH (ATP regeneration synthetic enzymes), thereby alleviating inflammation [196]. HMGB1 antagonists act as classic anti-DAMPs and have promising anti-inflammatory effects [197]. The use of coagulation factors, such as tranexamic acid, or blood transfusion strategies also enhances the efficacy of the management of TICs [198, 199]. New approaches for understanding the immune profile and the crosstalk with other organs affected by trauma, such as modulating regulatory T (Treg) cells [200], mesenchymal stem cells [201], macrophages [202], and their derived nanoparticles [203], are necessary to improve the management of trauma.

Although mechanistic studies have suggested therapeutic targets for trauma and other inflammatory organ failure-related diseases, a series of critical studies on the feasibility, potential risks, and clinical application of this novel treatment are urgently needed for translational purposes.

## Conclusions

Trauma is a severe systemic disease that occurs throughout a patient’s life due to organ inflammation and dysfunction. This review summarizes the different mechanisms of trauma-induced inflammation and organ dysfunction. In particular, DAMPs, trauma-induced cell death, organ–organ cross-talk pathways, and the gut microbiota play vital roles in the development of posttraumatic systemic inflammation. In addition, new approaches for trauma diagnosis, treatment, and prognosis are discussed. We can explore methods to protect against and improve treatment efficacy when trauma-induced inflammation and organ dysfunction occur.

## Abbreviations

**Table:** 

AIM2	Absent in melanoma 2
ALI	Acute lung injury
AQP4	Aquaporin 4
ATP	Adenosine 5′ triphosphate
ARDS	Acute respiratory distress syndrome
AKI	Acute kidney injury
ADAMTS13	Disintegrin-like and metalloprotease with thrombospondin type 1 motif 13
AGEs	Advanced glycation end products
AMPK	Adenosine 5′-monophosphate (AMP)-activated protein kinase
BAs	Bile acids
BBB	Blood–brain barrier
CT	Computed tomography
CCL2	C-C motif chemokine ligand 2
CCR2	C-C chemokine receptor type 2
CIRP	Cold-inducible RNA-binding protein
CXCL1	C-X-C motif ligand 1
Ca^2+^	Calcium
Cl^−^	Chloride
CaSR	Calcium-sensing receptor
cGAS	Cyclic guanosine monophosphate-adenosine monophosphate synthase
cDAMPs	Constitutive damps
CORM-3	Monoxide-releasing molecule-3
DAMPs	Damage-associated molecular patterns
DCs	Dendritic cells
EC	Endothelial cell
ETC	Electron transport chain
FPP	Farnesyl pyrophosphate
GPRC6A	G protein-coupled receptor family C group 6 member A
GPX4	Glutathione peroxidase 4
GGPP	Geranylgeranyl diphosphate
GSDMD	Gasdermin protein family
HSP	Heat shock protein
HS	Hemorrhagic shock
HMGB1	High mobility group box 1
HPA	Hypothalamic–pituitary–adrenal
HO-1	Heme oxygenase 1
IL-1β	Interleukin-1beta
IL-6	Interleukin-6
IL-12	Interleukin-12
iNKT	Intestinal natural killer T
ICAM-1	intracellular adhesion molecule-1
JNK	Jun N-terminal kinase
Keap1	Kelch-like ECH-associated protein 1
K^+^	Potassium
LPS	Lipopolysaccharide
MHCII	Histocompatibility complex class II
MODS	Multiple organ dysfunction syndrome
mtDNA	Mitochondrial DNAs
rmCIRP	Murine CIRP
MLVEC	Mouse lung vascular endothelial cell
MAPK	Mitogen-activated protein kinases
MCP-1	Monocyte chemoattractant protein-1
MyD88	Myeloid differentiation factor-88
MLKL	Mixed lineage kinase domain-like protein
miRNAs	MicroRNAs
MMPs	Matrix metalloproteinases
MC1R	Melanocortin 1 receptor
Mac-1	Macrophage-1 antigen 1
NF-κB	Nuclear factor-kappa B
NLRP3	NOD-like receptor pyrin domain-containing protein 3
NET	Neutrophil extracellular trap
Nrf2	Nuclear factor-erythroid factor 2-related factor 2
NADPH	Nicotinamide adenine dinucleotide phosphate
NO	Nitric oxide
OxLDLs	Oxidized low-density lipoproteins
OXPHOS	Oxidative phosphorylation
PF	Pseudo-fracture
PYD	Pyrin domain
PAMP	Pathogen-associated molecular pattern
PGC-1α	Peroxisome proliferator-activated receptor gamma coactivator 1-alpha
P2X7	Purine receptor 7
PINK1	PTEN-induced putative kinase 1
RAGE	Receptor for advanced glycation-end products
ROS	Reactive oxygen species
RIPK1	Receptor-interacting protein kinase
ST2	Suppression of tumorigenicity 2 receptor
SIRS	Systemic inflammatory response syndrome
SFAs	Saturated fatty acids
Sp1	Specificity protein 1
scRNA-seq	Single-cell RNA sequencing
SAH	Subarachnoid hemorrhage
SIRT1	Silent information regulator 1
TRIF	TIR domain-containing adaptor inducing IFN-beta
TBI	Traumatic brain injury
TLR2/4)	Toll-like receptors-2/4
TNF-α	Tumor Necrosis factor-Alpha
TLR9	Toll-like receptor 9
TIC	Trauma-induced coagulopathy
TF	Tissue factor
TAK1	Transforming growth factor-beta-activated kinase 1
TGF-β	Transforming growth factor-beta

## Data Availability

Not applicable.

## References

[1] Dogrul BN, Kiliccalan I, Asci ES, Peker SC. Blunt trauma related chest wall and pulmonary injuries: an overview. *Chin J Traumatol* 2020;23:125–38. 10.1016/j.cjtee.2020.04.003. 32417043 PMC7296362

[2] Lu R, Chotirosniramit N, Chandacham K, Jirapongcharoenlap T, Homchan OU, Kittidumkerng T. et al. Association between clinical factors and mortality in older adult trauma patients: a systematic review and meta-analysis. *Am J Surg* 2024;236:115890. 10.1016/j.amjsurg.2024.115890. 39153467

[3] Wray JP, Bridwell RE, Schauer SG, Shackelford SA, Bebarta VS, Wright FL. et al. The diamond of death: hypocalcemia in trauma and resuscitation. *Am J Emerg Med* 2021;41:104–9. 10.1016/j.ajem.2020.12.065. 33421674

[4] Schlapbach LJ, Watson RS, Sorce LR, Argent AC, Menon K, Hall MW. et al. International consensus criteria for pediatric sepsis and septic shock. *JAMA* 2024;331:665–74. 10.1001/jama.2024.0179. 38245889 PMC10900966

[5] Rosovsky R, Borges J, Kabrhel C, Rosenfield K. Pulmonary embolism response team: inpatient structure, outpatient follow-up, and is it the current standard of care? *Clin Chest Med* 2018;39:621–30. 10.1016/j.ccm.2018.04.019. 30122185

[6] Rosovsky R, Zhao K, Sista A, Rivera-Lebron B, Kabrhel C. Pulmonary embolism response teams: purpose, evidence for efficacy, and future research directions. *Res Pract Thromb Haemost* 2019;3:315–30. 10.1002/rth2.12216. 31294318 PMC6611377

[7] Ryckman FC, Noseworthy J. Multisystem trauma. *Surg Clin North Am* 1985;65:1287–302. 10.1016/s0039-6109(16)43740-0. 3904047

[8] Brazinova A, Rehorcikova V, Taylor MS, Buckova V, Majdan M, Psota M. et al. Epidemiology of traumatic brain injury in Europe: a living systematic review. *J Neurotrauma* 2021;38:1411–40. 10.1089/neu.2015.4126. 26537996 PMC8082737

[9] Wang XP, Zhong J, Lei T, Wang HJ, Zhu LN, Chu S. et al. Epidemiology of traumatic brain injury-associated epilepsy in western China: an analysis of multicenter data. *Epilepsy Res* 2020;164:106354. 10.1016/j.eplepsyres.2020.106354. 32438297

[10] Mata-Bermudez A, Trejo-Chavez R, Martinez-Vargas M, Perez-Arredondo A, Martinez-Cardenas MLA, Diaz-Ruiz A. et al. Dysregulation of the dopaminergic system secondary to traumatic brain injury: implications for mood and anxiety disorders. *Front Neurosci* 2024;18:1447688. 10.3389/fnins.2024.1447688. 39176379 PMC11338874

[11] Darmanto AG, Jan JS, Yen TL, Huang SW, Teng RD, Wang JY. et al. Targeting circadian protein rev-erbalpha to alleviate inflammation, oxidative stress, and enhance functional recovery following brain trauma. *Antioxidants (Basel)* 2024;13:901. 10.3390/antiox13080901. 39199147 PMC11351136

[12] Jones KA, Sadri S, Ahmad N, Weintraub JR, Reis SP. Thoracic trauma, nonaortic injuries. *Semin Intervent Radiol* 2021;38:75–83. 10.1055/s-0041-1726005. 33883804 PMC8049753

[13] Cakmak G, Cansun F, Saracoglu A, K TS. Airway management in penetrating thoracic trauma. *Anaesthesiol Intensive Ther* 2022;54:253–61. 10.5114/ait.2022.118332. 36000693 PMC10156565

[14] Ludwig C, Koryllos A. Management of chest trauma. *J Thorac Dis* 2017;9:S172–7. 10.21037/jtd.2017.03.52. 28446982 PMC5392544

[15] Coccolini F, Stahel PF, Montori G, Biffl W, Horer TM, Catena F. et al. Pelvic trauma: Wses classification and guidelines. *World J Emerg Surg* 2017;12:5. 10.1186/s13017-017-0117-6. 28115984 PMC5241998

[16] Okada N, Mitani H, Mori T, Ueda M, Chosa K, Fukumoto W. et al. Transarterial embolization to treat hemodynamically unstable trauma patients with splenic injuries: a retrospective multicenter observational study. *Injury* 2024;56:111768. 10.1016/j.injury.2024.111768. 39117521

[17] Durso AM, Paes FM, Caban K, Danton G, Braga TA, Sanchez A. et al. Evaluation of penetrating abdominal and pelvic trauma. *Eur J Radiol* 2020;130:109187. 10.1016/j.ejrad.2020.109187. 32745896

[18] Costantini TW, Coimbra R, Holcomb JB, Podbielski JM, Catalano R, Blackburn A. et al. Current management of hemorrhage from severe pelvic fractures: results of an American association for the surgery of trauma multi-institutional trial. *J Trauma Acute Care Surg* 2016;80:717–23. 10.1097/TA.0000000000001034. 26958799

[19] Loftus TJ, Morrow ML, Lottenberg L, Rosenthal MD, Croft CA, Smith RS. et al. The impact of prior laparotomy and intra-abdominal adhesions on bowel and mesenteric injury following blunt abdominal trauma. *World J Surg* 2019;43:457–65. 10.1007/s00268-018-4792-6. 30225563 PMC6330127

[20] McKinley TO, Gaski GE, Vodovotz Y, Corona BT, Billiar TR. Diagnosis and management of polytraumatized patients with severe extremity trauma. *J Orthop Trauma* 2018;32:S1–6. 10.1097/BOT.0000000000001114. 29461394

[21] Yang L, Xu Z, Liu J, Chang X, Ren Z, Xiao W. Multi-omics insights into bone tissue injury and healing: bridging orthopedic trauma and regenerative medicine. *Burns Trauma* 2025;13:tkaf019. 10.1093/burnst/tkaf019. 40438296 PMC12118463

[22] Kuwahara JT, Kord A, Ray CE Jr. Penetrating extremity trauma endovascular versus open repair? *Semin Intervent Radiol* 2020;37:55–61. 10.1055/s-0039-3401840. 32139971 PMC7056340

[23] Namas RA, Almahmoud K, Mi Q, Ghuma A, Namas R, Zaaqoq A. et al. Individual-specific principal component analysis of circulating inflammatory mediators predicts early organ dysfunction in trauma patients. *J Crit Care* 2016;36:146–53. 10.1016/j.jcrc.2016.07.002. 27546764 PMC5097026

[24] Rowe CJ, Mang J, Huang B, Dommaraju K, Potter BK, Schobel SA. et al. Systemic inflammation induced from remote extremity trauma is a critical driver of secondary brain injury. *Mol Cell Neurosci* 2023;126:103878. 10.1016/j.mcn.2023.103878. 37451414

[25] Billiar TR, Hunt BJ, Bailly S. Targeting inflammation in traumatic injury: entering a new era. *Intensive Care Med* 2023;49:977–8. 10.1007/s00134-023-07152-2. 37466669

[26] Matzinger P . Tolerance, danger, and the extended family. *Annu Rev Immunol* 1994;12:991–1045. 10.1146/annurev.iy.12.040194.005015. 8011301

[27] Land W . Allograft injury mediated by reactive oxygen species: from conserved proteins of drosophila to acute and chronic rejection of human transplants. Part III: interaction of (oxidative) stress-induced heat shock proteins with toll-like receptor-bearing cells of innate immunity and its consequences for the development of acute and chronic allograft rejection. *Transplant Rev* 2003;17:67–86. 10.1016/S0955-470X(02)00009-5.

[28] Shim YR, Jeong WI. Recent advances of sterile inflammation and inter-organ cross-talk in alcoholic liver disease. *Exp Mol Med* 2020;52:772–80. 10.1038/s12276-020-0438-5. 32457490 PMC7272465

[29] Ma M, Jiang W, Zhou R. Damps and damp-sensing receptors in inflammation and diseases. *Immunity* 2024;57:752–71. 10.1016/j.immuni.2024.03.002. 38599169

[30] Roh JS, Sohn DH. Damage-associated molecular patterns in inflammatory diseases. *Immune Netw* 2018;18:e27. 10.4110/in.2018.18.e27. 30181915 PMC6117512

[31] Kaur J, Singh H, Naqvi S. Intracellular damps in neurodegeneration and their role in clinical therapeutics. *Mol Neurobiol* 2023;60:3600–16. 10.1007/s12035-023-03289-9. 36859688

[32] Mahaling B, Low SWY, Beck M, Kumar D, Ahmed S, Connor TB. et al. Damage-associated molecular patterns (damps) in retinal disorders. *Int J Mol Sci* 2022;23:2591. 10.3390/ijms23052591. 35269741 PMC8910759

[33] She R, Liu D, Liao J, Wang G, Ge J, Mei Z. Mitochondrial dysfunctions induce panoptosis and ferroptosis in cerebral ischemia/reperfusion injury: from pathology to therapeutic potential. *Front Cell Neurosci* 2023;17:1191629. 10.3389/fncel.2023.1191629. 37293623 PMC10244524

[34] Piantadosi CA . Mitochondrial DNA, oxidants, and innate immunity. *Free Radic Biol Med* 2020;152:455–61. 10.1016/j.freeradbiomed.2020.01.013. 31958498

[35] Deng M, Scott MJ, Fan J, Billiar TR. Location is the key to function: Hmgb1 in sepsis and trauma-induced inflammation. *J Leukoc Biol* 2019;106:161–9. 10.1002/JLB.3MIR1218-497R. 30946496 PMC6597316

[36] Muire PJ, Schwacha MG, Wenke JC. Systemic t cell exhaustion dynamics is linked to early high mobility group box protein 1 (hmgb1) driven hyper-inflammation in a polytrauma rat model. *Cells* 2021;10:1646. 10.3390/cells10071646. 34209240 PMC8305113

[37] Sun Q, Loughran P, Shapiro R, Shrivastava IH, Antoine DJ, Li T. et al. Redox-dependent regulation of hepatocyte absent in melanoma 2 inflammasome activation in sterile liver injury in mice. *Hepatology* 2017;65:253–68. 10.1002/hep.28893. 27774630 PMC5191963

[38] Deng C, Zhao L, Yang Z, Shang JJ, Wang CY, Shen MZ. et al. Targeting hmgb1 for the treatment of sepsis and sepsis-induced organ injury. *Acta Pharmacol Sin* 2022;43:520–8. 10.1038/s41401-021-00676-7. 34040166 PMC8888646

[39] Chen R, Kang R, Tang D. The mechanism of hmgb1 secretion and release. *Exp Mol Med* 2022;54:91–102. 10.1038/s12276-022-00736-w. 35217834 PMC8894452

[40] Meevassana J, Jiraboonsri S, Jitworawisut A, Khayanying N, Sirimaharaj P, Kamolratanakul S. et al. Box a of hmgb1 improves second-degree burn wound healing in rats. *Burns* 2025;51:107456. 10.1016/j.burns.2025.107456. 40187206

[41] Chen S, Zhou A, Yan W. Hmgb1 promotes accelerated fracture healing in traumatic brain injury through pink1/parkin-mediated mitochondrial autophagy. *Biol Pharm Bull* 2024;47:2143–53. 10.1248/bpb.b24-00203. 39710383

[42] Rai V, Mathews G, Agrawal DK. Translational and clinical significance of damps, pamps, and prrs in trauma-induced inflammation. *Arch Clin Biomed Res* 2022;6:673–85. 10.26502/acbr.50170279. 36147548 PMC9491702

[43] Michetti F, Clementi ME, Di Liddo R, Valeriani F, Ria F, Rende M. et al. The s100b protein: a multifaceted pathogenic factor more than a biomarker. *Int J Mol Sci* 2023;24:9605. 10.3390/ijms24119605. 37298554 PMC10253509

[44] Muller M, Munster JM, Hautz WE, Gerber JL, Schefold JC, Exadaktylos AK. et al. Increased s-100 b levels are associated with fractures and soft tissue injury in multiple trauma patients. *Injury* 2020;51:812–8. 10.1016/j.injury.2020.03.012. 32192718

[45] Blais Lecuyer J, Mercier E, Tardif PA, Archambault PM, Chauny JM, Berthelot S. et al. S100b protein level for the detection of clinically significant intracranial haemorrhage in patients with mild traumatic brain injury: a subanalysis of a prospective cohort study. *Emerg Med J* 2021;38:285–9. 10.1136/emermed-2020-209583. 33355233 PMC7982939

[46] Zou Z, Li L, Li Q, Zhao P, Zhang K, Liu C. et al. The role of s100b/rage-enhanced adam17 activation in endothelial glycocalyx shedding after traumatic brain injury. *J Neuroinflammation* 2022;19:46. 10.1186/s12974-022-02412-2. 35148784 PMC8832692

[47] Kim JY, Kim JW, Yenari MA. Heat shock protein signaling in brain ischemia and injury. *Neurosci Lett* 2020;715:134642. 10.1016/j.neulet.2019.134642. 31759081 PMC6925329

[48] Haider T, Simader E, Gluck O, Ankersmit HJ, Heinz T, Hajdu S. et al. Systemic release of heat-shock protein 27 and 70 following severe trauma. *Sci Rep* 2019;9:9595. 10.1038/s41598-019-46034-w. 31270381 PMC6610099

[49] Fan H, Ding R, Liu W, Zhang X, Li R, Wei B. et al. Heat shock protein 22 modulates nrf1/tfam-dependent mitochondrial biogenesis and drp1-sparked mitochondrial apoptosis through ampk-pgc1alpha signaling pathway to alleviate the early brain injury of subarachnoid hemorrhage in rats. *Redox Biol* 2021;40:101856. 10.1016/j.redox.2021.101856. 33472123 PMC7816003

[50] Poluzzi C, Nastase MV, Zeng-Brouwers J, Roedig H, Hsieh LT, Michaelis JB. et al. Biglycan evokes autophagy in macrophages via a novel cd44/toll-like receptor 4 signaling axis in ischemia/reperfusion injury. *Kidney Int* 2019;95:540–62. 10.1016/j.kint.2018.10.037. 30712922

[51] Frevert CW, Felgenhauer J, Wygrecka M, Nastase MV, Schaefer L. Danger-associated molecular patterns derived from the extracellular matrix provide temporal control of innate immunity. *J Histochem Cytochem* 2018;66:213–27. 10.1369/0022155417740880. 29290139 PMC5958376

[52] Tang T, Lang X, Xu C, Wang X, Gong T, Yang Y. et al. Clics-dependent chloride efflux is an essential and proximal upstream event for nlrp3 inflammasome activation. *Nat Commun* 2017;8:202. 10.1038/s41467-017-00227-x. 28779175 PMC5544706

[53] Zhang Z, Wang H, Tao B, Shi X, Chen G, Ma H. et al. Attenuation of blood–brain barrier disruption in traumatic brain injury via inhibition of nkcc1 cotransporter: insights into the nf-kappab/nlrp3 signaling pathway. *J Neurotrauma* 2025;42:814–31. 10.1089/neu.2023.0580. 39879999

[54] Mohsin M, Tabassum G, Ahmad S, Ali S, Ali SM. The role of mitophagy in pulmonary sepsis. *Mitochondrion* 2021;59:63–75. 10.1016/j.mito.2021.04.009. 33894359

[55] Kang N, Ji Z, Li Y, Gao J, Wu X, Zhang X. et al. Metabolite-derived damage-associated molecular patterns in immunological diseases. *FEBS J* 2024;291:2051–67. 10.1111/febs.16902. 37432883

[56] O'Rourke SA, Neto NGB, Devilly E, Shanley LC, Fitzgerald HK, Monaghan MG. et al. Cholesterol crystals drive metabolic reprogramming and m1 macrophage polarisation in primary human macrophages. *Atherosclerosis* 2022;352:35–45. 10.1016/j.atherosclerosis.2022.05.015. 35667162

[57] Teng X, Brown J, Choi SC, Li W, Morel L. Metabolic determinants of lupus pathogenesis. *Immunol Rev* 2020;295:167–86. 10.1111/imr.12847. 32162304 PMC7180129

[58] Wang J, Liu J, Wang Y, Lin M, Tian W, Zhou L. et al. High glucose induces alternative activation of macrophages via pi3k/akt signaling pathway. *J Recept Signal Transduct Res* 2017;37:409–15. 10.1080/10799893.2017.1298131. 28292218

[59] Liang S, Lv ZT, Zhang JM, Wang YT, Dong YH, Wang ZG. et al. Necrostatin-1 attenuates trauma-induced mouse osteoarthritis and il-1beta induced apoptosis via hmgb1/tlr4/sdf-1 in primary mouse chondrocytes. *Front Pharmacol* 2018;9:1378. 10.3389/fphar.2018.01378. 30542285 PMC6277802

[60] Weng W, He Z, Ma Z, Huang J, Han Y, Feng Q. et al. Tufm lactylation regulates neuronal apoptosis by modulating mitophagy in traumatic brain injury. *Cell Death Differ* 2025;32:530–45. 10.1038/s41418-024-01408-0. 39496783 PMC11894137

[61] Yilmaz N, Yilmaz AF, Ilhan YS. Resveratrol ameliorates hepatic injury and modulates hepatic biomarkers of regeneration, apoptosis and survival in a rat model of blunt hepatic trauma. *Bratisl Lek Listy* 2020;121:512–5. 10.4149/BLL_2020_084. 32990006

[62] Yan Z, He JL, Guo L, Zhang HJ, Zhang SL, Zhang J. et al. Activation of caspase-12 at early stage contributes to cardiomyocyte apoptosis in trauma-induced secondary cardiac injury. *Sheng Li Xue Bao* 2017;69:367–77. 28825094

[63] Li B, Guan G, Mei L, Jiao K, Li H. Pathological mechanism of chondrocytes and the surrounding environment during osteoarthritis of temporomandibular joint. *J Cell Mol Med* 2021;25:4902–11. 10.1111/jcmm.16514. 33949768 PMC8178251

[64] Yuan J, Ofengeim D. A guide to cell death pathways. *Nat Rev Mol Cell Biol* 2024;25:379–95. 10.1038/s41580-023-00689-6. 38110635

[65] Van Opdenbosch N, Lamkanfi M. Caspases in cell death, inflammation, and disease. *Immunity* 2019;50:1352–64. 10.1016/j.immuni.2019.05.020. 31216460 PMC6611727

[66] Messer MP, Kellermann P, Weber SJ, Hohmann C, Denk S, Klohs B. et al. Silencing of fas, fas-associated via death domain, or caspase 3 differentially affects lung inflammation, apoptosis, and development of trauma-induced septic acute lung injury. *Shock* 2013;39:19–27. 10.1097/SHK.0b013e318277d856. 23247118

[67] Huan Y, Wu XQ, Chen T, Dou YN, Jia B, He X. et al. Necroptosis plays a crucial role in the exacerbation of retinal injury after blunt ocular trauma. *Neural Regen Res* 2023;18:922–8. 10.4103/1673-5374.353848. 36204864 PMC9700109

[68] Wang L, Zhang Y, Huang M, Yuan Y, Liu X. Rip3 in necroptosis: underlying contributions to traumatic brain injury. *Neurochem Res* 2024;49:245–57. 10.1007/s11064-023-04038-z. 37743445

[69] Gupta R, Kumari S, Tripathi R, Ambasta RK, Kumar P. Unwinding the modalities of necrosome activation and necroptosis machinery in neurological diseases. *Ageing Res Rev* 2023;86:101855. 10.1016/j.arr.2023.101855. 36681250

[70] Li Z, Fan EK, Liu J, Scott MJ, Li Y, Li S. et al. Cold-inducible rna-binding protein through tlr4 signaling induces mitochondrial DNA fragmentation and regulates macrophage cell death after trauma. *Cell Death Dis* 2017;8:e2775. 10.1038/cddis.2017.187. 28492546 PMC5584526

[71] Li Z, Scott MJ, Fan EK, Li Y, Liu J, Xiao G. et al. Tissue damage negatively regulates lps-induced macrophage necroptosis. *Cell Death Differ* 2016;23:1428–47. 10.1038/cdd.2016.21. 26943325 PMC5072421

[72] Broz P, Pelegrin P, Shao F. The gasdermins, a protein family executing cell death and inflammation. *Nat Rev Immunol* 2020;20:143–57. 10.1038/s41577-019-0228-2. 31690840

[73] Frank D, Vince JE. Pyroptosis versus necroptosis: similarities, differences, and crosstalk. *Cell Death Differ* 2019;26:99–114. 10.1038/s41418-018-0212-6. 30341423 PMC6294779

[74] Bi F, Ma H, Ji C, Chang C, Liu W, Xie K. Rhein protects against neurological deficits after traumatic brain injury in mice via inhibiting neuronal pyroptosis. *Front Pharmacol* 2020;11:564367. 10.3389/fphar.2020.564367. 33101024 PMC7554525

[75] Ge X, Li W, Huang S, Yin Z, Xu X, Chen F. et al. The pathological role of nlrs and aim2 inflammasome-mediated pyroptosis in damaged blood–brain barrier after traumatic brain injury. *Brain Res* 2018;1697:10–20. 10.1016/j.brainres.2018.06.008. 29886252

[76] Yang WL, Sharma A, Wang Z, Li Z, Fan J, Wang P. Cold-inducible rna-binding protein causes endothelial dysfunction via activation of nlrp3 inflammasome. *Sci Rep* 2016;6:26571. 10.1038/srep26571. 27217302 PMC4877585

[77] Yang J, Zhao Y, Zhang P, Li Y, Yang Y, Yang Y. et al. Hemorrhagic shock primes for lung vascular endothelial cell pyroptosis: role in pulmonary inflammation following lps. *Cell Death Dis* 2016;7:e2363. 10.1038/cddis.2016.274. 27607578 PMC5059873

[78] Ming T, Yuan M, Kong Q, Huang Q, Xia Z, Wu X. Dexmedetomidine alleviates blunt chest trauma and hemorrhagic shock-resuscitation-induced acute lung injury through inhibiting the nlrp3 inflammasome. *Mol Med Rep* 2020;22:2507–15. 10.3892/mmr.2020.11335. 32705267 PMC7411430

[79] Zhang LM, Zhang DX, Zheng WC, Hu JS, Fu L, Li Y. et al. Corm-3 exerts a neuroprotective effect in a rodent model of traumatic brain injury via the bidirectional gut-brain interactions. *Exp Neurol* 2021;341:113683. 10.1016/j.expneurol.2021.113683. 33711325

[80] Shao XF, Li B, Shen J, Wang QF, Chen SS, Jiang XC. et al. Ghrelin alleviates traumatic brain injury-induced acute lung injury through pyroptosis/nf-kappab pathway. *Int Immunopharmacol* 2020;79:106175. 10.1016/j.intimp.2019.106175. 31918060

[81] Yu H, Guo P, Xie X, Wang Y, Chen G. Ferroptosis, a new form of cell death, and its relationships with tumourous diseases. *J Cell Mol Med* 2017;21:648–57. 10.1111/jcmm.13008. 27860262 PMC5345622

[82] Hambright WS, Fonseca RS, Chen L, Na R, Ran Q. Ablation of ferroptosis regulator glutathione peroxidase 4 in forebrain neurons promotes cognitive impairment and neurodegeneration. *Redox Biol* 2017;12:8–17. 10.1016/j.redox.2017.01.021. 28212525 PMC5312549

[83] Zhang Y, Fan BY, Pang YL, Shen WY, Wang X, Zhao CX. et al. Neuroprotective effect of deferoxamine on erastininduced ferroptosis in primary cortical neurons. *Neural Regen Res* 2020;15:1539–45. 10.4103/1673-5374.274344. 31997820 PMC7059591

[84] Hao J, Li B, Duan HQ, Zhao CX, Zhang Y, Sun C. et al. Mechanisms underlying the promotion of functional recovery by deferoxamine after spinal cord injury in rats. *Neural Regen Res* 2017;12:959–68. 10.4103/1673-5374.208591. 28761430 PMC5514872

[85] Chu C, Wang X, Yang C, Chen F, Shi L, Xu W. et al. Neutrophil extracellular traps drive intestinal microvascular endothelial ferroptosis by impairing fundc1-dependent mitophagy. *Redox Biol* 2023;67:102906. 10.1016/j.redox.2023.102906. 37812880 PMC10579540

[86] Altman AM, Miller MJ, Mahmud J, Smith NA, Chan GC. Human cytomegalovirus-induced autophagy prevents necroptosis of infected monocytes. *J Virol* 2020;94:e01022-20. 10.1128/JVI.01022-20. 32878887 PMC7592208

[87] Wu C, Chen H, Zhuang R, Zhang H, Wang Y, Hu X. et al. Betulinic acid inhibits pyroptosis in spinal cord injury by augmenting autophagy via the ampk-mtor-tfeb signaling pathway. *Int J Biol Sci* 2021;17:1138–52. 10.7150/ijbs.57825. 33867836 PMC8040310

[88] Xu Y, Hu X, Li F, Zhang H, Lou J, Wang X. et al. Gdf-11 protects the traumatically injured spinal cord by suppressing pyroptosis and necroptosis via tfe3-mediated autophagy augmentation. *Oxidative Med Cell Longev* 2021;2021:8186877. 10.1155/2021/8186877. PMC8548157 34712387

[89] Xu Y, Geng Y, Wang H, Zhang H, Qi J, Li F. et al. Cyclic helix b peptide alleviates proinflammatory cell death and improves functional recovery after traumatic spinal cord injury. *Redox Biol* 2023;64:102767. 10.1016/j.redox.2023.102767. 37290302 PMC10267601

[90] Zhang L, Lin Y, Bai W, Sun L, Tian M. Human umbilical cord mesenchymal stem cell-derived exosome suppresses programmed cell death in traumatic brain injury via pink1/parkin-mediated mitophagy. *CNS Neurosci Ther* 2023;29:2236–58. 10.1111/cns.14159. 36890626 PMC10352888

[91] Gando S, Levi M, Toh CH. Trauma-induced innate immune activation and disseminated intravascular coagulation. *J Thromb Haemost* 2024;22:337–51. 10.1016/j.jtha.2023.09.028. 37816463

[92] Gando S, Otomo Y. Local hemostasis, immunothrombosis, and systemic disseminated intravascular coagulation in trauma and traumatic shock. *Crit Care* 2015;19:72. 10.1186/s13054-015-0735-x. 25886801 PMC4337317

[93] Moore EE, Moore HB, Kornblith LZ, Neal MD, Hoffman M, Mutch NJ. et al. Trauma-induced coagulopathy. *Nat Rev Dis Primers* 2021;7:30. 10.1038/s41572-021-00264-3. 33927200 PMC9107773

[94] Grover SP, Mackman N. Tissue factor: An essential mediator of hemostasis and trigger of thrombosis. *Arterioscler Thromb Vasc Biol* 2018;38:709–25. 10.1161/ATVBAHA.117.309846. 29437578

[95] Russell RT, McDaniel JK, Cao W, Shroyer M, Wagener BM, Zheng XL. et al. Low plasma adamts13 activity is associated with coagulopathy, endothelial cell damage and mortality after severe paediatric trauma. *Thromb Haemost* 2018;118:676–87. 10.1055/s-0038-1636528. 29618154 PMC6433136

[96] Jin J, Wang F, Tian J, Zhao X, Dong J, Wang N. et al. Neutrophil extracellular traps contribute to coagulopathy after traumatic brain injury. *JCI*. *Insight.* 2023;8:e141110. 10.1172/jci.insight.141110. PMC10070118 36802340

[97] Hellenthal KEM, Brabenec L, Wagner NM. Regulation and dysregulation of endothelial permeability during systemic inflammation. *Cells.* 2022;11:1935. 10.3390/cells11121935. 35741064 PMC9221661

[98] Schoenfeld L, Appl B, Pagerols-Raluy L, Heuer A, Reinshagen K, Boettcher M. Immunofluorescence imaging of neutrophil extracellular traps in human and mouse tissues. *J Vis Exp* 2023;198:e65272. 10.3791/65272. 37677039

[99] Goswami J, MacArthur TA, Mahony C, Kizhakkedathu JN, Vappala S, Smith S. et al. Dnase-mediated dissolution of neutrophil extracellular traps accelerates in vitro thrombin generation kinetics in trauma patients. *Shock* 2022;58:217–23. 10.1097/SHK.0000000000001972. 35959777 PMC9810375

[100] MacArthur TA, Goswami J, Navarro SM, Spears GM, Bailey KR, Thompson R. et al. A murine multiple-injury model for the study of thromboinflammation. *J Trauma Acute Care Surg* 2024;96:203–8. 10.1097/TA.0000000000004179. 37934621 PMC10872879

[101] Alsabani M, Abrams ST, Cheng Z, Morton B, Lane S, Alosaimi S. et al. Reduction of netosis by targeting cxcr1/2 reduces thrombosis, lung injury, and mortality in experimental human and murine sepsis. *Br J Anaesth* 2022;128:283–93. 10.1016/j.bja.2021.10.039. 34893315 PMC8792833

[102] Strogulski NR, Portela LV, Polster BM, Loane DJ. Fundamental neurochemistry review: microglial immunometabolism in traumatic brain injury. *J Neurochem* 2023;167:129–53. 10.1111/jnc.15959. 37759406 PMC10655864

[103] Li M, Wang S, Zhang C, Chi C, Liu R, Wang T. et al. Escin alleviates stress-induced intestinal dysfunction to protect brain injury by regulating the gut-brain axis in ischemic stroke rats. *Int Immunopharmacol* 2023;115:109659. 10.1016/j.intimp.2022.109659. 36608442

[104] Taheri S, Karaca Z, Mehmetbeyoglu E, Hamurcu Z, Yilmaz Z, Dal F. et al. The role of apoptosis and autophagy in the hypothalamic–pituitary–adrenal (hpa) axis after traumatic brain injury (tbi). *Int J Mol Sci* 2022;23:15699. 10.3390/ijms232415699. 36555341 PMC9778890

[105] Xu W, Yan J, Ocak U, Lenahan C, Shao A, Tang J. et al. Melanocortin 1 receptor attenuates early brain injury following subarachnoid hemorrhage by controlling mitochondrial metabolism via ampk/sirt1/pgc-1alpha pathway in rats. *Theranostics* 2021;11:522–39. 10.7150/thno.49426. 33391490 PMC7738864

[106] Wu J, Cyr A, Gruen DS, Lovelace TC, Benos PV, Das J. et al. Lipidomic signatures align with inflammatory patterns and outcomes in critical illness. *Nat Commun* 2022;13:6789. 10.1038/s41467-022-34420-4. 36357394 PMC9647252

[107] Obermayer G, Afonyushkin T, Binder CJ. Oxidized low-density lipoprotein in inflammation-driven thrombosis. *J Thromb Haemost* 2018;16:418–28. 10.1111/jth.13925. 29316215

[108] Goodwin ML, Rothberg DL. Lactate metabolism in trauma. *J Trauma Acute Care Surg* 2014;77:182–3. 10.1097/TA.0000000000000162. 24977778

[109] Zadorozny EV, Weigel T, Stone A, Gruen DS, Galvagno SM Jr, Yazer MH. et al. Prehospital lactate is associated with the need for blood in trauma. *Prehosp Emerg Care* 2022;26:590–9. 10.1080/10903127.2021.1983096. 34550050

[110] Dienel GA . Lactate shuttling and lactate use as fuel after traumatic brain injury: metabolic considerations. *J Cereb Blood Flow Metab* 2014;34:1736–48. 10.1038/jcbfm.2014.153. 25204393 PMC4269761

[111] Patet C, Suys T, Carteron L, Oddo M. Cerebral lactate metabolism after traumatic brain injury. *Curr Neurol Neurosci Rep* 2016;16:31. 10.1007/s11910-016-0638-5. 26898683

[112] Capizzi A, Woo J, Verduzco-Gutierrez M. Traumatic brain injury: An overview of epidemiology, pathophysiology, and medical management. *Med Clin North Am* 2020;104:213–38. 10.1016/j.mcna.2019.11.001. 32035565

[113] Schantz SL, Sneed SE, Fagan MM, Golan ME, Cheek SR, Kinder HA. et al. Human-induced pluripotent stem cell-derived neural stem cell therapy limits tissue damage and promotes tissue regeneration and functional recovery in a pediatric piglet traumatic-brain-injury model. *Biomedicines* 2024;12:1663. 10.3390/biomedicines12081663. 39200128 PMC11351842

[114] Cash A, Theus MH. Mechanisms of blood–brain barrier dysfunction in traumatic brain injury. *Int J Mol Sci* 2020;21:3344. 10.3390/ijms21093344. 32397302 PMC7246537

[115] Mira RG, Quintanilla RA, Cerpa W. Mild traumatic brain injury induces mitochondrial calcium overload and triggers the upregulation of nclx in the hippocampus. *Antioxidants (Basel)* 2023;12:403. 10.3390/antiox12020403. 36829963 PMC9952386

[116] de Castro MRT, Ferreira APO, Busanello GL, da Silva LRH, da Silveira Junior MEP, Fiorin FDS. et al. Previous physical exercise alters the hepatic profile of oxidative-inflammatory status and limits the secondary brain damage induced by severe traumatic brain injury in rats. *J Physiol* 2017;595:6023–44. 10.1113/JP273933. 28726269 PMC5577552

[117] Kumar Sahel D, Kaira M, Raj K, Sharma S, Singh S. Mitochondrial dysfunctioning and neuroinflammation: recent highlights on the possible mechanisms involved in traumatic brain injury. *Neurosci Lett* 2019;710:134347. 10.1016/j.neulet.2019.134347. 31229625

[118] Yan L, Gu L, Lv X, Ni Z, Qian W, Chen Z. et al. Butylphthalide mitigates traumatic brain injury by activating anti-ferroptotic ahr-cyp1b1 pathway. *J Ethnopharmacol* 2024;337:118758. 10.1016/j.jep.2024.118758. 39222762

[119] Navabi SP, Badreh F, Khombi Shooshtari M, Hajipour S, Moradi Vastegani S, Khoshnam SE. Microglia-induced neuroinflammation in hippocampal neurogenesis following traumatic brain injury. *Heliyon* 2024;10:e35869. 10.1016/j.heliyon.2024.e35869. 39220913 PMC11365414

[120] Li H, Sun D, Zhao Z, Fang J, Li M, Lv C. et al. Neutrophil membrane-derived nanoparticles protect traumatic brain injury via inhibiting calcium overload and scavenging ros. *J Nanobiotechnology* 2024;22:477. 10.1186/s12951-024-02753-5. 39135044 PMC11320991

[121] Puntambekar SS, Saber M, Lamb BT, Kokiko-Cochran ON. Cellular players that shape evolving pathology and neurodegeneration following traumatic brain injury. *Brain Behav Immun* 2018;71:9–17. 10.1016/j.bbi.2018.03.033. 29601944

[122] Thapa K, Khan H, Singh TG, Kaur A. Traumatic brain injury: mechanistic insight on pathophysiology and potential therapeutic targets. *J Mol Neurosci* 2021;71:1725–42. 10.1007/s12031-021-01841-7. 33956297

[123] Zhang Y, Shen X, Deng S, Chen Q, Xu B. Neural regulation of vascular development: molecular mechanisms and interactions. *Biomolecules* 2024;14:966. 10.3390/biom14080966. 39199354 PMC11353022

[124] Corrigan F, Mander KA, Leonard AV, Vink R. Neurogenic inflammation after traumatic brain injury and its potentiation of classical inflammation. *J Neuroinflammation* 2016;13:264. 10.1186/s12974-016-0738-9. 27724914 PMC5057243

[125] Markou A, Kitchen P, Aldabbagh A, Repici M, Salman MM, Bill RM. et al. Mechanisms of aquaporin-4 vesicular trafficking in mammalian cells. *J Neurochem* 2024;168:100–14. 10.1111/jnc.16029. 38102893 PMC10953025

[126] Salman MM, Sheilabi MA, Bhattacharyya D, Kitchen P, Conner AC, Bill RM. et al. Transcriptome analysis suggests a role for the differential expression of cerebral aquaporins and the mapk signalling pathway in human temporal lobe epilepsy. *Eur J Neurosci* 2017;46:2121–32. 10.1111/ejn.13652. 28715131

[127] Martinez-Torres AM, Moran J. Aquaporin 4 and the endocannabinoid system: a potential therapeutic target in brain injury. *Exp Brain Res* 2024;242:2041–58. 10.1007/s00221-024-06896-7. 39043897 PMC11306651

[128] O'Neil DA, Nicholas MA, Lajud N, Kline AE, Bondi CO. Preclinical models of traumatic brain injury: emerging role of glutamate in the pathophysiology of depression. *Front Pharmacol* 2018;9:579. 10.3389/fphar.2018.00579. 29910733 PMC5992468

[129] Gruenbaum BF, Zlotnik A, Fleidervish I, Frenkel A, Boyko M. Glutamate neurotoxicity and destruction of the blood–brain barrier: key pathways for the development of neuropsychiatric consequences of tbi and their potential treatment strategies. *Int J Mol Sci* 2022;23:9628. 10.3390/ijms23179628. 36077024 PMC9456007

[130] Lin CJ, Chen TH, Yang LY, Shih CM. Resveratrol protects astrocytes against traumatic brain injury through inhibiting apoptotic and autophagic cell death. *Cell Death Dis* 2014;5:e1147. 10.1038/cddis.2014.123. 24675465 PMC3973229

[131] Di Pietro V, Yakoub KM, Caruso G, Lazzarino G, Signoretti S, Barbey AK. et al. Antioxidant therapies in traumatic brain injury. *Antioxidants (Basel)* 2020;9:260. 10.3390/antiox9030260. 32235799 PMC7139349

[132] Gadani SP, Walsh JT, Lukens JR, Kipnis J. Dealing with danger in the cns: the response of the immune system to injury. *Neuron.* 2015;87:47–62. 10.1016/j.neuron.2015.05.019. 26139369 PMC4491143

[133] Corps KN, Roth TL, McGavern DB. Inflammation and neuroprotection in traumatic brain injury. *JAMA Neurol* 2015;72:355–62. 10.1001/jamaneurol.2014.3558. 25599342 PMC5001842

[134] Castellanos-Molina A, Bretheau F, Boisvert A, Belanger D, Lacroix S. Constitutive damps in cns injury: from preclinical insights to clinical perspectives. *Brain Behav Immun* 2024;122:583–95. 10.1016/j.bbi.2024.07.047. 39222725

[135] Boraschi D, Italiani P, Weil S, Martin MU. The family of the interleukin-1 receptors. *Immunol Rev* 2018;281:197–232. 10.1111/imr.12606. 29248002

[136] Visser K, Koggel M, Blaauw J, van der Horn HJ, Jacobs B, van der Naalt J. Blood-based biomarkers of inflammation in mild traumatic brain injury: a systematic review. *Neurosci Biobehav Rev* 2022;132:154–68. 10.1016/j.neubiorev.2021.11.036. 34826510

[137] Fan H, Tang HB, Chen Z, Wang HQ, Zhang L, Jiang Y. et al. Inhibiting hmgb1-rage axis prevents pro-inflammatory macrophages/microglia polarization and affords neuroprotection after spinal cord injury. *J Neuroinflammation* 2020;17:295. 10.1186/s12974-020-01973-4. 33036632 PMC7547440

[138] Wang W, Pang C, Zhang J, Peng L, Zhang X, Shi L. et al. Takinib inhibits microglial m1 polarization and oxidative damage after subarachnoid hemorrhage by targeting tak1-dependent nlrp3 inflammasome signaling pathway. *Front Immunol* 2023;14:1266315. 10.3389/fimmu.2023.1266315. 38035075 PMC10682771

[139] Martin NT, Martin MU. Interleukin 33 is a guardian of barriers and a local alarmin. *Nat Immunol* 2016;17:122–31. 10.1038/ni.3370. 26784265

[140] Wang Y, Fu WY, Cheung K, Hung KW, Chen C, Geng H. et al. Astrocyte-secreted il-33 mediates homeostatic synaptic plasticity in the adult hippocampus. *Proc Natl Acad Sci USA* 2021;118:e2020810118. 10.1073/pnas.2020810118. 33443211 PMC7817131

[141] Nguyen PT, Dorman LC, Pan S, Vainchtein ID, Han RT, Nakao-Inoue H. et al. Microglial remodeling of the extracellular matrix promotes synapse plasticity. *Cell* 2020;182:e15. 10.1016/j.cell.2020.05.050. PMC7497728 32615087

[142] Faroqi AH, Lim MJ, Kee EC, Lee JH, Burgess JD, Chen R. et al. In vivo detection of extracellular adenosine triphosphate in a mouse model of traumatic brain injury. *J Neurotrauma* 2021;38:655–64. 10.1089/neu.2020.7226. 32935624 PMC7898407

[143] Yorkgitis BK, Tatum DM, Taghavi S, Schroeppel TJ, Noorbakhsh MR, Philps FH. et al. Eastern association for the surgery of trauma multicenter trial: comparison of pre-injury antithrombotic use and reversal strategies among severe traumatic brain injury patients. *J Trauma Acute Care Surg* 2022;92:88–92. 10.1097/TA.0000000000003421. 34570064

[144] Pan P, Song Y, Du X, Bai L, Hua X, Xiao Y. et al. Intestinal barrier dysfunction following traumatic brain injury. *Neurol Sci* 2019;40:1105–10. 10.1007/s10072-019-03739-0. 30771023

[145] Bortolotti P, Faure E, Kipnis E. Inflammasomes in tissue damages and immune disorders after trauma. *Front Immunol* 2018;9:1900. 10.3389/fimmu.2018.01900. 30166988 PMC6105702

[146] Kerr NA, de Rivero Vaccari JP, Weaver C, Dietrich WD, Ahmed T, Keane RW. Enoxaparin attenuates acute lung injury and inflammasome activation after traumatic brain injury. *J Neurotrauma* 2021;38:646–54. 10.1089/neu.2020.7257. 32669032 PMC7898405

[147] Qian Y, Gao C, Zhao X, Song Y, Luo H, An S. et al. Fingolimod attenuates lung injury and cardiac dysfunction after traumatic brain injury. *J Neurotrauma* 2020;37:2131–40. 10.1089/neu.2019.6951. 32434456

[148] El-Menyar A, Goyal A, Latifi R, Al-Thani H, Frishman W. Brain-heart interactions in traumatic brain injury. *Cardiol Rev* 2017;25:279–88. 10.1097/CRD.0000000000000167. 28984668

[149] Dimitri GM, Beqiri E, Placek MM, Czosnyka M, Stocchetti N, Ercole A. et al. Modeling brain-heart crosstalk information in patients with traumatic brain injury. *Neurocrit Care* 2022;36:738–50. 10.1007/s12028-021-01353-7. 34642842 PMC9110542

[150] Biso S, Wongrakpanich S, Agrawal A, Yadlapati S, Kishlyansky M, Figueredo V. A review of neurogenic stunned myocardium. *Cardiovasc Psychiatry Neurol* 2017;2017:5842182. 10.1155/2017/5842182. 28875040 PMC5569748

[151] Plott C, Harb T, Arvanitis M, Gerstenblith G, Blumenthal R, Leucker T. Neurocardiac axis physiology and clinical applications. *Int J Cardiol Heart Vasc* 2024;54:101488. 10.1016/j.ijcha.2024.101488. 39224460 PMC11367645

[152] Zhao Q, Yan T, Li L, Chopp M, Venkat P, Qian Y. et al. Immune response mediates cardiac dysfunction after traumatic brain injury. *J Neurotrauma* 2019;36:619–29. 10.1089/neu.2018.5766. 30045672

[153] Khandelwal M, Krishna G, Ying Z, Gomez-Pinilla F. Liver acts as a metabolic gate for the traumatic brain injury pathology: protective action of thyroid hormone. *Biochim Biophys Acta Mol basis Dis* 2023;1869:166728. 10.1016/j.bbadis.2023.166728. 37137432 PMC10601893

[154] Nizamutdinov D, DeMorrow S, McMillin M, Kain J, Mukherjee S, Zeitouni S. et al. Hepatic alterations are accompanied by changes to bile acid transporter-expressing neurons in the hypothalamus after traumatic brain injury. *Sci Rep* 2017;7:40112. 10.1038/srep40112. 28106051 PMC5247752

[155] Palafox-Sanchez V, Ying Z, Royes LFF, Gomez-Pinilla F. The interaction between brain and liver regulates lipid metabolism in the tbi pathology. *Biochim Biophys Acta Mol basis Dis* 2021;1867:166078. 10.1016/j.bbadis.2021.166078. 33444711 PMC7889047

[156] Yuan H, Tian Y, Jiang R, Wang Y, Nie M, Li X. et al. Susceptibility to hepatotoxic drug-induced liver injury increased after traumatic brain injury in mice. *J Neurotrauma* 2024;41:1425–37. 10.1089/neu.2022.0147. 37265124

[157] Hanscom M, Loane DJ, Shea-Donohue T. Brain-gut axis dysfunction in the pathogenesis of traumatic brain injury. *J Clin Invest* 2021;131:e143777. 10.1172/JCI143777. 34128471 PMC8203445

[158] Revell MA, Pugh MA, McGhee M. Gastrointestinal traumatic injuries: gastrointestinal perforation. *Crit Care Nurs Clin North Am* 2018;30:157–66. 10.1016/j.cnc.2017.10.014. 29413211

[159] Sundman MH, Chen NK, Subbian V, Chou YH. The bidirectional gut-brain-microbiota axis as a potential nexus between traumatic brain injury, inflammation, and disease. *Brain Behav Immun* 2017;66:31–44. 10.1016/j.bbi.2017.05.009. 28526435

[160] Pathare N, Sushilkumar S, Haley L, Jain S, Osier NN. The impact of traumatic brain injury on microbiome composition: a systematic review. *Biol Res Nurs* 2020;22:495–505. 10.1177/1099800420943961. 32720519

[161] Skrifvars MB, Bailey M, Moore E, Martensson J, French C, Presneill J. et al. A post hoc analysis of osmotherapy use in the erythropoietin in traumatic brain injury study-associations with acute kidney injury and mortality. *Crit Care Med* 2021;49:e394–403. 10.1097/CCM.0000000000004853. 33566466 PMC7963441

[162] Lee S, Hwang H, Yamal JM, Goodman JC, Aisiku IP, Gopinath S. et al. Impact probability of poor outcome and plasma cytokine concentrations are associated with multiple organ dysfunction syndrome following traumatic brain injury. *J Neurosurg* 2019;131:1931–7. 10.3171/2018.8.JNS18676. 30641838

[163] Civiletti F, Assenzio B, Mazzeo AT, Medica D, Giaretta F, Deambrosis I. et al. Acute tubular injury is associated with severe traumatic brain injury: In vitro study on human tubular epithelial cells. *Sci Rep* 2019;9:6090. 10.1038/s41598-019-42147-4. 30988316 PMC6465296

[164] McDonald SJ, Sharkey JM, Sun M, Kaukas LM, Shultz SR, Turner RJ. et al. Beyond the brain: peripheral interactions after traumatic brain injury. *J Neurotrauma* 2020;37:770–81. 10.1089/neu.2019.6885. 32041478

[165] Gao MZ, Zeng JY, Chen XJ, Shi L, Hong FY, Lin M. et al. Dimethyl fumarate ameliorates oxidative stress-induced acute kidney injury after traumatic brain injury by activating keap1-nrf2/ho-1 signaling pathway. *Heliyon* 2024;10:e32377. 10.1016/j.heliyon.2024.e32377. 38947486 PMC11214498

[166] Chan BD, Wong WY, Lee MM, Cho WC, Yee BK, Kwan YW. et al. Exosomes in inflammation and inflammatory disease. *Proteomics* 2019;19:e1800149. 10.1002/pmic.201800149. 30758141

[167] Anand S, Samuel M, Kumar S, Mathivanan S. Ticket to a bubble ride: cargo sorting into exosomes and extracellular vesicles. *Biochim Biophys Acta, Proteins Proteomics* 2019;1867:140203. 10.1016/j.bbapap.2019.02.005. 30822540

[168] Kalluri R, McAndrews KM. The role of extracellular vesicles in cancer. *Cell* 2023;186:1610–26. 10.1016/j.cell.2023.03.010. 37059067 PMC10484374

[169] Console L, Scalise M, Indiveri C. Exosomes in inflammation and role as biomarkers. *Clin Chim Acta* 2019;488:165–71. 10.1016/j.cca.2018.11.009. 30419221

[170] Mahesh G, Biswas R. Microrna-155: a master regulator of inflammation. *J Interf Cytokine Res* 2019;39:321–30. 10.1089/jir.2018.0155. PMC6591773 30998423

[171] Li P, Zhao R, Fan K, Iwanowycz S, Fan H, Li Z. et al. Regulation of dendritic cell function improves survival in experimental sepsis through immune chaperone. *Innate Immun* 2019;25:235–43. 10.1177/1753425919840423. 31018807 PMC6830886

[172] Walsh SA, Hoyt BW, Rowe CJ, Dey D, Davis TA. Alarming cargo: the role of exosomes in trauma-induced inflammation. *Biomolecules* 2021;11:522. 10.3390/biom11040522. 33807302 PMC8065643

[173] Murao A, Brenner M, Aziz M, Wang P. *Exosomes in sepsis Front Immunol* 2020;11:2140. 10.3389/fimmu.2020.02140. 33013905 PMC7509534

[174] Jiao Y, Li Z, Loughran PA, Fan EK, Scott MJ, Li Y. et al. Frontline science: macrophage-derived exosomes promote neutrophil necroptosis following hemorrhagic shock. *J Leukoc Biol* 2018;103:175–83. 10.1189/jlb.3HI0517-173R. 28801344 PMC6346432

[175] Wang C, Li Q, Ren J. Microbiota-immune interaction in the pathogenesis of gut-derived infection. *Front Immunol* 2019;10:1873. 10.3389/fimmu.2019.01873. 31456801 PMC6698791

[176] Chen P, Billiar T. Gut microbiota and multiple organ dysfunction syndrome (mods). *Adv Exp Med Biol* 2020;1238:195–202. 10.1007/978-981-15-2385-4_11. 32323186

[177] Liu YJ, Tang B, Wang FC, Tang L, Lei YY, Luo Y. et al. Parthenolide ameliorates colon inflammation through regulating treg/th17 balance in a gut microbiota-dependent manner. *Theranostics* 2020;10:5225–41. 10.7150/thno.43716. 32373209 PMC7196297

[178] Wang Y, Yin Y, Chen X, Zhao Y, Wu Y, Li Y. et al. Induction of intestinal th17 cells by flagellins from segmented filamentous bacteria. *Front Immunol* 2019;10:2750. 10.3389/fimmu.2019.02750. 31824516 PMC6883716

[179] Brennan CA, Clay SL, Lavoie SL, Bae S, Lang JK, Fonseca-Pereira D. et al. Fusobacterium nucleatum drives a pro-inflammatory intestinal microenvironment through metabolite receptor-dependent modulation of il-17 expression. *Gut Microbes* 2021;13:1987780. 10.1080/19490976.2021.1987780. 34781821 PMC8604392

[180] Yuan B, Lu XJ, Wu Q. Gut microbiota and acute central nervous system injury: a new target for therapeutic intervention. *Front Immunol* 2021;12:800796. 10.3389/fimmu.2021.800796. 35003127 PMC8740048

[181] Yang Y, Xu C, Wu D, Wang Z, Wu P, Li L. et al. Gammadelta t cells: crosstalk between microbiota, chronic inflammation, and colorectal cancer. *Front Immunol* 2018;9:1483. 10.3389/fimmu.2018.01483. 29997627 PMC6028700

[182] Amaral WZ, Kokroko N, Treangen TJ, Villapol S, Gomez-Pinilla F. Probiotic therapy modulates the brain-gut-liver microbiota axis in a mouse model of traumatic brain injury. *Biochim Biophys Acta Mol basis Dis* 2024;1870:167483. 10.1016/j.bbadis.2024.167483. 39209236 PMC11526848

[183] Lee KC, Chen P, Maricic I, Inamine T, Hu J, Gong S. et al. Intestinal inkt cells migrate to liver and contribute to hepatocyte apoptosis during alcoholic liver disease. *Am J Physiol Gastrointest Liver Physiol* 2019;316:G585–97. 10.1152/ajpgi.00269.2018. 30817180 PMC6580241

[184] Cheng XC, Tong WZ, Rui W, Feng Z, Shuai H, Zhe W. Single-cell sequencing technology in skin wound healing. *Burns*. *Trauma* 2024;12:tkae043. 10.1093/burnst/tkae043. PMC11497848 39445224

[185] Chen T, Delano MJ, Chen K, Sperry JL, Namas RA, Lamparello AJ. et al. A road map from single-cell transcriptome to patient classification for the immune response to trauma. *JCI Insight* 2021;6:e145108. 10.1172/jci.insight.145108. 33320841 PMC7934885

[186] Fu G, Chen T, Wu J, Jiang T, Tang D, Bonaroti J. et al. Single-cell transcriptomics reveals compartment-specific differences in immune responses and contributions for complement factor 3 in hemorrhagic shock plus tissue trauma. *Shock* 2021;56:994–1008. 10.1097/SHK.0000000000001765. 33710107 PMC8429528

[187] Hu X, Xu W, Ren Y, Wang Z, He X, Huang R. et al. Spinal cord injury: molecular mechanisms and therapeutic interventions. *Signal Transduct Target Ther* 2023;8:245. 10.1038/s41392-023-01477-6. 37357239 PMC10291001

[188] Cai M, Yue M, Chen T, Liu J, Forno E, Lu X. et al. Robust and accurate estimation of cellular fraction from tissue omics data via ensemble deconvolution. *Bioinformatics* 2022;38:3004–10. 10.1093/bioinformatics/btac279. 35438146 PMC9991889

[189] Chen T, Wei Y, Vodovotz Y, Chen W, Billiar TR. Longitudinal analysis of transcriptomic subtypes in trauma patients. *Shock* 2022;58:34–7. 10.1097/SHK.0000000000001958. 35904142 PMC9391314

[190] Chen T, Conroy J, Wang X, Situ M, Namas RA, Vodovotz Y. et al. The independent prognostic value of global epigenetic alterations: An analysis of single-cell atac-seq of circulating leukocytes from trauma patients followed by validation in whole blood leukocyte transcriptomes across three etiologies of critical illness. *EBioMedicine* 2022;76:103860. 10.1016/j.ebiom.2022.103860. 35124428 PMC8822299

[191] Li SR, Moheimani H, Herzig B, Kail M, Krishnamoorthi N, Wu J. et al. High-dimensional proteomics identifies organ injury patterns associated with outcomes in human trauma. *J Trauma Acute Care Surg* 2023;94:803–13. 10.1097/TA.0000000000003880. 36787435 PMC10205666

[192] Shuwari N, Inoue C, Ishigami I, Jingushi K, Kamiya M, Kawakami S. et al. Small extracellular vesicles carrying reovirus, tumor antigens, interferon-beta, and damage-associated molecular patterns for efficient tumor treatment. *J Control Release* 2024;374:89–102. 10.1016/j.jconrel.2024.07.079. 39122217

[193] Hollis R, Tenet M, Aziz M, Wang P. Anti-damp therapies for acute inflammation. *Front Immunol* 2025;16:1579954. 10.3389/fimmu.2025.1579954. 40406124 PMC12094975

[194] Yang H, Wang H, Andersson U. Targeting inflammation driven by hmgb1. *Front Immunol* 2020;11:484. 10.3389/fimmu.2020.00484. 32265930 PMC7099994

[195] Nofi CP, Wang P, Aziz M. Chromatin-associated molecular patterns (camps) in sepsis. *Cell Death Dis* 2022;13:700. 10.1038/s41419-022-05155-3. 35961978 PMC9372964

[196] Jia YJ, Xiong S, Yao M, Wei Y, He Y. Hmgb1 inhibition blocks ferroptosis and oxidative stress to ameliorate sepsis-induced acute lung injury by activating the nrf2 pathway. *Kaohsiung J Med Sci* 2024;40:710–21. 10.1002/kjm2.12851. 38837857 PMC11895617

[197] Land WG . Use of damps and samps as therapeutic targets or therapeutics: a note of caution. *Mol Diagn Ther* 2020;24:251–62. 10.1007/s40291-020-00460-z. 32248387 PMC7127836

[198] James A, Cole E, Dunser M, Bouzat P, Gauss T. Acute traumatic coagulopathy: what you should know, what is debated and what should come next. *Anaesth Crit Care Pain Med* 2025;44:101543. 10.1016/j.accpm.2025.101543. 40383226

[199] Brunskill SJ, Disegna A, Wong H, Fabes J, Desborough MJ, Doree C. et al. Blood transfusion strategies for major bleeding in trauma. *Cochrane Database Syst Rev* 2025;4:CD012635. 10.1002/14651858.CD012635.pub2. 40271704 PMC12019925

[200] Anvarinia Y, Del Mar NA, Seetharaman ATM, Hossain MS, Prislovsky A, Mandal N. et al. The essential role of immunomodulatory tregs in visual deficits after military-relevant trauma. *Mil Med* 2025;190:e2404-e2412. 10.1093/milmed/usaf254. PMC12588709 40478534

[201] Zhao Y, Ye C, Wang H, Chen C, Lu Y, Yang C. et al. Loading tea polyphenols enhances the repair of human umbilical cord mesenchymal stem cell sheet after spinal cord injury. *Stem Cell Res Ther* 2025;16:264. 10.1186/s13287-025-04376-5. 40437527 PMC12121034

[202] Bahader GA, Warner T, Hall MW, Sribnick EA. Granulocyte-macrophage colony-stimulating factor reduces lung bacterial load following traumatic brain injury and hemorrhage polytrauma in a juvenile rat model. *PLoS One* 2025;20:e0323674. 10.1371/journal.pone.0323674. 40388470 PMC12088021

[203] Tian L, Jin J, Lai F, Yao S, Zhang Y, Liu J. et al. Nebulized m2 macrophage-derived nanovesicles for the treatment of explosion-induced acute lung injury. *J Colloid Interface Sci* 2025;691:137381. 10.1016/j.jcis.2025.137381. 40187079

